# Potential Effects of MSC-Derived Exosomes in Neuroplasticity in Alzheimer’s Disease

**DOI:** 10.3389/fncel.2018.00317

**Published:** 2018-09-24

**Authors:** Edwin E. Reza-Zaldivar, Mercedes A. Hernández-Sapiéns, Benito Minjarez, Yanet K. Gutiérrez-Mercado, Ana L. Márquez-Aguirre, Alejandro A. Canales-Aguirre

**Affiliations:** ^1^Unidad de Evaluación Preclínica, Biotecnología Médica y Farmacéutica, CONACYT Centro de Investigación y Asistencia en Tecnología y Diseño del Estado de Jalisco (CIATEJ), Guadalajara, Mexico; ^2^Centro Universitario de Ciencias Biológicas y Agropecuarias (CUCBA), Universidad de Guadalajara, Guadalajara, Mexico; ^3^Profesor del programa de Maestría en Ciencias de la Salud Ambiental, Centro Universitario de Ciencias Biológicas y Agropecuarias (CUCBA), Universidad de Guadalajara, Guadalajara, Mexico

**Keywords:** exosomes, Alzheimer’s disease, neuroplasticity, exosomal cargo, proteomics, miRNA

## Abstract

Alzheimer’s disease (AD) is the most common type of dementia affecting regions of the central nervous system that exhibit synaptic plasticity and are involved in higher brain functions such as learning and memory. AD is characterized by progressive cognitive dysfunction, memory loss and behavioral disturbances of synaptic plasticity and energy metabolism. Cell therapy has emerged as an alternative treatment of AD. The use of adult stem cells, such as neural stem cells and Mesenchymal Stem Cells (MSCs) from bone marrow and adipose tissue, have the potential to decrease cognitive deficits, possibly by reducing neuronal loss through blocking apoptosis, increasing neurogenesis, synaptogenesis and angiogenesis. These processes are mediated primarily by the secretion of many growth factors, anti-inflammatory proteins, membrane receptors, microRNAs (miRNA) and exosomes. Exosomes encapsulate and transfer several functional molecules like proteins, lipids and regulatory RNA which can modify cell metabolism. In the proteomic characterization of the content of MSC-derived exosomes, more than 730 proteins have been identified, some of which are specific cell type markers and others are involved in the regulation of binding and fusion of exosomes with adjacent cells. Furthermore, some factors were found that promote the recruitment, proliferation and differentiation of other cells like neural stem cells. Moreover, within exosomal cargo, a wide range of miRNAs were found, which can control functions related to neural remodeling as well as angiogenic and neurogenic processes. Taking this into consideration, the use of exosomes could be part of a strategy to promote neuroplasticity, improve cognitive impairment and neural replacement in AD. In this review, we describe how exosomes are involved in AD pathology and discuss the therapeutic potential of MSC-derived exosomes mediated by miRNA and protein cargo.

## Introduction

Alzheimer’s disease (AD) is characterized by the progressive deposition of β-amyloid (Aβ) around neurons and the intracellular accumulation of neurofibrillary tangles (NFT) of hyperphosphorylated tau, mainly in areas implicated in memory and learning, such as the prefrontal cortex and hippocampus. In advanced stages of the disease, aggregates of Aβ are present in motor areas, cerebrospinal fluid, as well as in eyes and neuromuscular joints (Reiss et al., 2018).

Presently there is no effective treatment for AD hence, stem cell therapy has been proposed to be a promising therapeutic option for this neurological disorder. Cell therapies for brain restoration generally target multiple cells of the brain parenchyma such as endothelial cells, neural stem cells (also named neural progenitors) and oligodendrocyte precursor cells. The interaction between the administered cells and resident cells promote neuroplastic events such angiogenesis stimulation, neurogenesis and axonal remodeling, result in a neurological recovery (Xin et al., 2017a; Xiong et al., 2017).

Several studies have demonstrated the effectiveness of Mesenchymal Stem Cells (MSCs) treatment in several neurodegenerative diseases (Wei et al., 2013). These cells have typical stem cell characteristics like the potential to differentiate into multiple cell lineages under different physiological conditions, including the ability to selectively migrate towards damage sites (homing) and interact with brain parenchyma cells. This interaction stimulate the production of neurotrophins such as vascular endothelial growth factor (VEGF), hepatocyte growth factor (HGF), nerve growth factor (NGF), brain-derived neurotrophic factor (BDNF) and neurotrophin-3 (Li et al., 2002; Kurozumi et al., 2004; Kim et al., 2010; Matthay et al., 2017) which increase neuritic development, promote neurorestoration and neurological recovery (Xiong et al., 2017; Harting et al., 2018).

Among the main functions of MSCs are their ability to limit inflammation environments through the release of soluble factors such as HGF, prostaglandin E2, transforming growth factor β1, indoleamine 2,3 dioxygenase, interleukin 10 and nitric oxide. This immunomodulatory environment allows the expression of growth factors, high immunomodulatory protein secretion and the enhancement of endogenous cellular repair processes (Nguyen et al., 2013; Phinney and Pittenger, 2017).

A central hypothesis has been proposed, in which MSCs are implied to exert a dynamic homeostatic response that supports tissue preservation as well as function recovery (Harting et al., 2018). The main mechanism by which MSCs mediate this activity is not the cellular implant and its subsequent differentiation, but the paracrine activity of the secretome (Nakano et al., 2016; Yang Y. et al., 2017). This phenomenon was demonstrated in studies where conditioned medium of MSCs was administered and therapeutic effects similar to those already reported for MSCs were produced in different animal models of diseases (Timmers et al., 2007; Mitsialis and Kourembanas, 2016). A subsequent fractionation of this conditioned medium was performed and an active component of approximately 50–150 nm was found. Biophysical studies categorized these compounds as exosomes (Lai et al., 2010; Phinney and Pittenger, 2017). Consequently, it was established that one of the critical parameters that regulate the paracrine activity of MSC is the generation of exosomes (Drommelschmidt et al., 2017; Phinney and Pittenger, 2017). Therefore, exosomes may be a therapeutic option in the treatment of AD because they exert therapeutic effects like MSCs.

### Biogenesis of Exosomes

Exosomes are small (30–150 nm diameter) membrane-enclosed vesicles of endosomal origin, released by a variety of cell types, capable of transferring biologically active macromolecules, such as proteins, lipids and RNA, to other cells (Bang and Thum, 2012). Exosomes are originated as intraluminal vesicles within the multivesicular bodies (MVB) by inward budding of the late endosomal membrane (Colombo et al., 2014). The Endosomal Sorting Complex Required for Transport (ESCRT) machinery is important in this process. ESCRT consist of approximately 20 proteins that assemble four different complexes; ESCRT-0, -I, -II, -III and the associated AAA ATPase vacuolar protein sorting 34 (Vps4) complex (Henne et al., 2013). ESCRT-0 recognizes and sequesters ubiquitylated proteins in the endosomal membrane, ESCRT-I and -II are responsible for membrane budding as well as recruiting of ESCRT-III that finally drive vesicle scission (Hurley and Hanson, 2010). The dissociation and recycling of the ESCRTs require the AAA ATPase Vps4 complex. Transport of MVB towards plasma membrane depends on interaction with the cytoskeleton, this interaction is mediated mainly by Rab GTPases and SNARE proteins, although precise mechanism of action in this process is not known (Ostrowski et al., 2009; Beer and Wehman, 2017). MVB subsequently fuse with the plasma membrane and release those intraluminal vesicles such as exosomes (Camacho et al., 2013; Abels and Breakefield, 2016). Some studies also suggest that MVB biogenesis can occur without ESCRTs. It has been shown that despite simultaneously silencing key subunits of all four ESCRTs, intraluminal vesicles are still formed in MVB, indicating the presence of a mechanism independent of ESCRT (Stuffers et al., 2009). Tetraspanins (Escola et al., 1998) and lipids (mainly ceramide; Trajkovic et al., 2008) could be essential players in exosome biogenesis due to the formation of microdomains that coalescence into larger domains that promote membrane budding.

As mentioned above, exosomes contain different proteins, lipids and nucleic acids (DNA, mRNA, microRNAs (miRNA), lncRNA), however, determining the exact composition and content of the exosomal content (cargo) produced by different cell types is hard to establish due to differences in the conditions which the cells are found. It should be mentioned that cellular homeostasis is an important factor that controls exosome cargo and secretion, therefore the exosomes will present characteristics that reflect its cellular origin (de Jong et al., 2012; Harting et al., 2018). Mechanisms for sorting cargo molecules into exosomes are still poorly understood. However, the ubiquitination is considered the main sorting signal for protein cargo entry into exosomes. Ubiquitinated proteins are recognized by receptors such as ESCRT subunits responsible for binding and directing cargo towards intraluminal vesicles (Piper and Katzmann, 2007). Usually these vesicles contain proteins that are involved in its biogenesis mechanisms, for example, ESCRT system components such as tetraspanins CD63, CD81 and CD9, as well as ALIX, TSG10, likewise proteins associated with their secretion as RAB27A, RAB11 and ARF6 (Wu et al., 2015; Abels and Breakefield, 2016). There are different pathways for miRNA sorting, which include: (I) neutral sphingomyelinase 2 pathway demonstrated by Kosaka et al. (2010), in where they found that overexpression of neutral sphingomyelinase 2 increased the amount of miRNA into exosomes, while its chemical inhibition reduced the number of miRNAs; (II) the miRNA motif and sumoylated heterogeneous nuclear ribonucleoproteins (hnRNPs) pathway reported by Villarroya-Beltri et al. (2013), identified a short sequence motifs in miRNAs (GGAG) in the portion 3′ that is recognized by exosomal sumoylated hnRNPs, this hnRNP-miRNA binding control the miRNA loading into exosomes; (III) the miRNA induced silencing complex (miRSC) pathway. Components of miRSC include miRNA, miRNA repressible mRNA, and proteins GW182 and AGO2; Guduric-Fuchs et al. (2012) discovered that knockout of AGO2 decreases the abundance of miRNA exported by exosomes. Besides AGO2, others components of miRSC like GW182 were found to be colocalized with MVB (Guduric-Fuchs et al., 2012). Despite this evidence of exosomal cargo sorting, the underlying mechanisms remain unclear.

Concerning lipid composition of the exosomal membrane, there are some lipids such as sphingomyelin, cholesterol, ganglioside GM3, phosphatidylserine and ceramide that form lipid raft domains that are more abundant in the exosomal membrane than in the cell of origin (Angeloni et al., 2016). In contrast, phosphatidylcholine and diacylglycerol are scarce in the membrane of exosomes compared to the cell membrane (Abels and Breakefield, 2016).

### Exosomes as Intercellular Communication Mediators

There is evidence suggesting that exosomes are internalized into recipient cells (Mulcahy et al., 2014). However, elucidation of the mechanisms of exosome targeting and uptake by recipient cells remains an important challenge. Exosomes could bear combinations of ligands that would engage different cell-surface receptors simultaneously, therefore different mechanisms have been proposed by which a cell can interact and uptake these nanovesicles. This communication could be through membrane receptors and the subsequent exosome membrane fusion with the cell membrane to exchange proteins and cytosol components. An other mechanism is through endocytosis, among which are clathrin-mediated endocytosis, caveolin-mediated endocytosis (Svensson et al., 2013), phagocytosis mediated mainly by phosphatidylserine, and micropinocytosis. The uptake mechanism used may depend on proteins and glycoproteins found on the surface of both the nanovesicle and the target cell.

Different studies establish that exosomes are mediators of intercellular communication, since they reach biological fluids such as blood, cerebrospinal fluid and urine among others, and act as paracrine messengers through the transference of bioactive lipids, mRNAs, miRNA, lncRNAs, and can also transfer genomic DNA and mitochondrial DNA and different proteins (Kalra et al., 2012; Keerthikumar et al., 2016). This transference of bioactive molecules establishing cell-cell communication processes can in an epigenetic way, alter the activity of the cells both in physiological and pathological conditions (Xiong et al., 2017; Harting et al., 2018).

Interestingly, the evidence shows that exosomes are released more under pathological conditions (Cheng et al., 2017). In this way, the most studied pathogenic components that use exosomes as infection route are the prion proteins (Vella et al., 2008), responsible for transmissible neurodegenerative diseases such as bovine spongiform encephalopathy and α-synuclein (Emmanouilidou et al., 2010), involved in Parkinson’s disease pathology. Prion diseases are fatal neurodegenerative disorders associated with the conversion of the cellular prion protein into the scrapie prion protein, an abnormal conformational state that tends to form amyloid deposits in brain tissue leading to dementia (Vingtdeux et al., 2012). On the other hand, exosomes released from cells that have an overproduction of α-synuclein can transfer this protein to normal cells and promote the overproduction by alterations in the ESCRT system that result in an increased exocytosis of exosomes with α-synuclein (Spencer et al., 2016). In AD, it has been proposed that exosomes have a key pathological function in the progression of the disease, and are involved in Aβ and tau dissemination, since an accumulation of exosomes has been found in amyloid plaques (Rajendran et al., 2006) and hyperphosphorylated tau tangles (Saman et al., 2012, 2014; Levy, 2017).

## Alzheimer’s Disease

AD is the most common neurodegenerative disease characterized by neuron loss and impairment of memory, cognition and functions of daily living. In many cases, death results from the loss of fine motor skills and incapacitation (Koelsch, 2017; Mroczko et al., 2018). The main pathological markers of AD are the accumulation of Aβ plaques and the formation of NFT, composed of hyperphosphorylated tau protein (Eitan et al., 2016). In early stages, these pathological changes are primarily localized within the medial temporal lobe and are spread through the neocortex (Braak and Braak, 1996).

Accumulation of Aβ in oligomers is one of the earliest events in the disease process, occurring 10–20 years prior to the onset of memory loss and other clinical symptoms (Reiman et al., 2012). Amyloid plaque formation are the result of Aβ peptides deposition that takes place in early endosomes, this process involves sequential hydrolysis of the amyloid precursor protein (APP) by β and γ-secretases (Rajendran et al., 2006). The β-site APP cleaving enzyme 1 (BACE1) is a transmembrane type I aspartyl protease that is located in endosomes as an immature precursor protein, and later in lysosomes and Golgi complex as a mature protein that catalyzes the initial amyloidogenic cleavage at β-site of APP while the membrane-associated 99 amino acid carboxyl-terminal fragment β remains (Munro et al., 2016; Yan et al., 2016). The γ-secretase has been identified as a multimeric protein complex containing presenilin 1, presenilin 2 associated with nicastrin, Aph-1 and Pen-2. The carboxyl-terminal fragment β is cleaved by γ-secretase releasing Aβ peptides (Sharples et al., 2008). The Aβ peptides released have pathophysiological impacts on synaptic function through inhibition of transmission of the synaptic signal leading neuronal death (Mroczko et al., 2018).

On the other hand, NFTs are formed by massive accumulations of abnormal insoluble polymers, referred to as paired helical filaments (Wischik et al., 1985, 1988). The main structural component of this filaments is tau, a microtubule-associated protein (Kosik et al., 1986). The physiological function of tau is to stabilize microtubules in the cell cytoskeleton, an activity regulated by its phosphorylation (Grundke-Iqbal et al., 1986). It has been suggested that abnormal phosphorylation is an early molecular event that may lead to a sequence of structural changes in the tau molecule, such as conformational changes like truncations (Luna-Muñoz et al., 2007) and is thought that hyperphosphorylation and its aggregation are related to the disassembling of neuronal microtubules, that consequently affect axonal transport and result in cell death (Stoothoff and Johnson, 2005). Hyperphosphorylation of tau primarily occurs at Ser-Pro or Thr-Pro motifs, suggesting that proline-directed kinases such as the MAPK, GSK3β and CDK5 are directly involved (Mandelkow et al., 1992; Baumann et al., 1993; Greenberg et al., 1994). Other kinases are also able to modify the tau molecule, including CAMK, PKA and PKC (Correas et al., 1992; Scott et al., 1993; Ghosh and Giese, 2015).

Dissemination of Aβ and tau has been suggested to be mediated through release of extracellular vesicles (EVs; Nath et al., 2012). EV are small membrane vesicles which result from the budding of the plasma membrane as microvesicles (also called ectosomes) or from the exocytosis of MVB as exosomes. EV is considered one of the distant extracellular communication agents due to its capacity to carry and deliver different types of components to target cells (Zhang and Yang, 2018). A relationship between EV and progression of AD has been proposed because most of the Aβ and tau oligomers are colocalized with late endosome/lysosome markers, mainly MVB (Nath et al., 2012; Joshi et al., 2015). During disease progression, both these histopathological hallmarks extend throughout the brain with characteristic patterns reaching limbic and association areas (Cho et al., 2016).

### Role of Exosomes in Alzheimer’s Disease

Although the origin of the disease remains unknown, several investigations have postulated prion-like mechanisms in AD progression and dissemination, including direct cell communication through gap junctions, synaptic transmission and exacerbated paracrine signaling due to alterations of endosomal/lysosomal secretion system, in which exosomes play a fundamental role in the distribution of neuropathological components between neuronal cells (Gauthier et al., 2017; Xiao et al., 2017; Laulagnier et al., 2018).

Subcellular location of neuronal Aβ was identified using immunoelectron microscopy by Takahashi et al. (2002), they found that Aβ42 is localized predominantly within MVB of the neurons. Accumulation of Aβ inside neurons is prevented by autophagy, an event occurring in the endosomal/lysosomal system where Aβ within endosomes are destroyed by lysosomes (Mizushima and Komatsu, 2011). A key regulator of this system is phosphatidylinositol-3-phosphate (PI3P), a phospholipid synthesized mainly by class III PI3-kinase Vps34 (Jaber et al., 2016). Miranda et al. (2018) showed that disruption of neuronal Vps34 (a retromer complex component) function impairs autophagy, lysosomal degradation as well as lipid metabolism. This promotes the secretion of unique exosomes enriched with undigested lysosomal substrates, including Aβ, APP and the enzymes that process APP in an amyloidogenic way (Malm et al., 2016). In addition, this accumulation increases with aging and it is associated with abnormal synaptic morphology (Takahashi et al., 2002). Overall, inhibiting neutral sphingomyelinase 2, a key regulatory enzyme in ceramide synthesis and exosome biogenesis, reduced the number of exosomes in the brain and serum and further reduced Aβ plaque load in 5×FAD mice (Dinkins et al., 2016). These observations suggest that MVB is essential for APP metabolism and Aβ secretion (Takahashi et al., 2002; Joshi et al., 2015). Furthermore, other studies demonstrated that transference of damaged neuronal cell-derived exosomes with APP, γ/β secretases, Aβ peptides, APP-CTF, ubiquitins, modified ubiquitin ligases and tau protein to adjacent neurons can lead to AD propagation (Chen et al., 2017; Yuyama and Igarashi, 2017; Zheng et al., 2017; Miranda et al., 2018).

An interactome analysis demonstrated that inhibition of γ-secretase activity results in a significant increase of exosomes enriched with APP-CTF suggesting the association of γ-secretase in exosome membrane. Also, it was shown that exosomes tetraspanins CD9 and CD81 interact with the γ-secretase complex regulating their activity in a positive way. Using neutralizing antibodies against CD9 and CD81 result in the disruption of Aβ generation and lead to an accumulation of the APP-CTF (Wakabayashi et al., 2009). Likewise, tetraspanin 6 enrichment in exosomal membrane allows the accumulation of Aβ, CTF-APP and BACE1 in exosomes, and independently of ESCRT, increases biogenesis of exosomes and secretion of this type of cargo, as well as inhibits the degradation of these nanovesicles by the lysosomal system (Guix et al., 2017). Thereby, these studies suggest the involvement of the tetraspanin web protein in the up and down regulation of Aβ generation.

It has been reported that the endosomal localization of BACE1 is regulated by the ACG sequence and the retromer, a multiprotein complex required for the recycling of transmembrane proteins from the endosomes to the trans-Golgi network (Tan and Evin, 2012). Kizuka et al. (2015) showed that BACE1 is modified with bisecting N-acetylglucosamine, a sugar modification highly expressed in the brain of AD patients, by GnT-III. They reported that lack of this modification directs BACE1 to late/lysosomes where it is less colocalized with APP, however, the glycan modification is protective for lysosomal degradation.

Furthermore, the Aβ peptides already present in extracellular space can interact with the exosomal membrane through their glycosphingolipids and the cellular prion protein (PrP^C^), forming aggregates of Aβ (Rajendran et al., 2006; Zappulli et al., 2016; Yuyama and Igarashi, 2017; Zheng et al., 2017). This was demonstrated in the histological analysis performed in brains of AD patients were an enrichment of exosomal markers Alix and flotillin-1 was found around neuritic plaques; this suggested that exosomes function as nucleation centers for amyloid plaque formation (Xiao et al., 2017). A recent publication by Falker et al. (2016) showed that PrP^C^ is highly enriched on exosomes membranes and distinct Aβ oligomers bind PrP^C^ with high affinity via its flexible N-terminus. This bind drives Aβ fibrillation and may be involved in the extracellular deposition of Aβ. However, there is a debate about if PrP^C^ is required for Aβ-mediated synaptotoxicity and suppression of long-term potentiation (Lauren et al., 2009; Kessels et al., 2010).

On the other hand, it has been proposed that the spread of tau can occur through neuronal synaptic connections, but the mechanism underlying this process remains unknown (Wang Y. et al., 2017). However, it also has been reported that monomers and oligomers of tau hyperphosphorylated are encapsulated within the exosomes (Shi et al., 2016), which are then transferred through synaptic contact with other neurons, and like the exosomes that interact with Aβ, can promote nucleation centers for hyperphosphorylated tau aggregation (Saman et al., 2012, 2014).

In addition to neural cell interaction, exosomes from damaged cells also interact with glial cells. Consequently, astrocytes not only fail to support neurons but also generate a toxic environment that is detrimental to neurons and astrocytes themselves through promoting secondary apoptosis of adjacent cells (Wang et al., 2012). Wang et al. (2012) found that the astrocytic-mediated apoptosis is associated with the secretion of PAR-4/ceramide containing exosomes in the adjacent cells even if they were not exposed to Aβ. It has been demonstrated that astrocytes tend to interact more with exosomes and accumulate large amounts of Aβ42 protofibers, subsequently, this storage results in endosomal/lysosomal system alterations which induce exosome secretion with a neurotoxic cargo (Nikitidou et al., 2017). Astrocyte-derived exosomes of patients with AD had up to 20-fold higher concentrations of β/γ-secretase and sAPPβ than neuron-derived exosomes (Goetzl et al., 2016). Moreover, Chiarini et al., 2017 presented evidence showing that tau and its hyperphosphorylated form are expressed by untransformed astrocytes in culture exposed to Aβ, the release is mediated by exosomes to the extracellular medium.

In addition, microglia also participates in the internalization of exosomes derived from damaged cells, Ikezu et al. (2016) found that microglia transduces tau aggregates into nearby neuronal cells via exosome secretion, tau aggregates propagate from cortical neurons to dentate granular cells and this propagation is sensitive to exosome inhibition or microglial depletion. In AD, Aβ phagocytosis by microglia is one of the principal mechanisms for a level decrease of these peptides. Exosome phagocytosis is a process mediated by phosphatidylserine; as well as in apoptotic cells, exosomal phosphatidylserine is found in the outer layer of the membrane, so it can be recognized by microglia phosphatidylserine receptor (Yuyama and Igarashi, 2017). However, in AD, microglia activity is markedly diminished, therefore, when Aβ interacts with exosomes, it initiates the formation of large aggregates in the form of plaques (Zheng et al., 2017).

Since AD has a long asymptomatic latency period, many investigators are searching for biomarkers that can detect the disease early on, particularly in its pre-symptomatic and early stages. Different studies show that deregulation in miRNA expression and its traffic via exosomes has repercussions on AD pathogenesis (Lugli et al., 2015). miRNAs are endogenous, short, noncoding RNAs of 18–25 nucleotides which act as important post-transcriptional regulators of gene expression by binding with their target mRNA (Liu C. G. et al., 2014). Currently there are about 2,650 different miRNAs identified in all human tissues and only 34–40 miRNA are abundant in the brain (Jaber et al., 2017), among them, there are different miRNAs that bind specifically to key genes that determine the expression of APP and β-secretase, such as miR-193b, miR-101 and miR-29c respectively, these miRNAs negatively influence the generation of Aβ (Lei et al., 2015; Chen et al., 2017). Nevertheless, it has been found that expression of these miRNAs decreased with AD progression (Liu C. G. et al., 2014). Lugli et al. (2015) performed an exosomal miRNAs analysis samples of people with AD and control people. They indicated that 20 miRNAs showed differential expression in AD, and miR-342-3p, miR-141-3p, miR-342-5p, miR-23b-3p, miR-24-3p, miR-125b-5p and miR-152-3p were selected as most predictive for AD group identity. Furthermore, miR-9, miR-125b, miR-191-5p, miR-181c and let-7g-5p are thought to be the best candidates for early biomarkers (Trotta et al., 2018).

As mentioned above, defects in protein transport are closely related with neurodegeneration. In this context, it has been reported that genes like SEC22B and SEC63 which participate in protein transport and regulation of cell motion are downregulated by miR-206 in the AD, the increase of this miRNA leads to a disequilibrium of proteostasis in the brain that could result in Aβ accumulation (Zhao et al., 2016b).

On the other hand, it has been shown that the miR-132/miR-212 cluster regulates tau expression. Smith et al. (2015) showed that miR-132/miR-212 deficiency in mice leads to increased tau expression, phosphorylation and aggregation, an effect associated with an autophagy dysfunction. Conversely, treatment of AD mice with miR-132/miR-212 restore, in part, memory dysfunction and tau metabolism.

Some miRNAs like miR-139 over express in AD, this overexpression impairs the hippocampus-dependent learning and memory formation by targeting the cannabinoid receptor type 2 (Tang et al., 2017), a membrane marker of activated microglial cells, which triggers pathophysiological events involved in synaptic plasticity and neuroprotection but is also implicated in diverse roles in regulating memory, depending on memory types and brain areas (Li and Kim, 2016).

However, due to the high degree of heterogeneity in miRNAs, further in-depth investigation is required to provide easily identifiable biomarkers of AD that can be isolated from blood or its components. It is also important to consider is the possibility of using miRNA approaches like the modulation of these miRNAs for the treatment of AD.

## Focus on MSC-Derived Exosomes and Their Role in Neuroplasticity

MSCs have multipotent mesodermal differentiation potential, but more importantly, they have demonstrated the ability to promote tissue repair through the release of paracrine factors, mainly a variety of growth factors, immunomodulatory cytokines and other trophic mediators, which make them an attractive therapeutic strategy for applications in inflammatory and chronic-degenerative diseases (Donders et al., 2018). In general, administration of MSCs or conditioned media from MSCs induce structural and functional benefits that reduce apoptosis at the lesion site, module proinflammatory response, provide a permissive environment for axonal extension, enhance neurogenesis and ameliorate neurological deficits (Cantinieaux et al., 2013; Qu and Zhang, 2017; Harris et al., 2018).

The composition of exosomal cargo determines the therapeutic potential of exosomes, and the fact that these vesicles were produced by cells with a therapeutic activity already described (like MSCs), increases this potential. Besides, MSCs are the most efficient exosome producing cells (Hall et al., 2016). Based on these facts and the paracrine hypothesis which establishes that the beneficial effect of stem cell therapy is due to stimulation of resident cell by secretion of bioactive molecules and release of EV, the use of exosomes could offer several advantages over MSCs such as a superior safety profile. Since these vesicles do not replicate they are exempted from uncontrolled division, unlike MSC, which during its isolation and expansion there is a risk of genetic damage which can lead to proliferation issues and spontaneous differentiation promoting tumor formation. Furthermore, exosomes lack metabolism, so the environment where they are administered will have no impact, also, they have a nanometric size, which decreases the possibility of microvascular thrombotic events, they can be sterilized by filtration, can be stored for long periods without presenting functional loss, and above all, have similar effects to those that MSCs exert with no side effects (Nakano et al., 2016; Ophelders et al., 2016; Gomzikova and Rizvanov, 2017; Xiong et al., 2017).

Many studies have shown that exosomes derived from MSCs can reduce cognitive problems associated with various neurological disorders models such as Traumatic Brain Injury (TBI; Xiong et al., 2017), Parkinson’s disease and stroke (Yang Y. et al., 2017). It has been hypothesized that these vesicles act as paracrine activity effectors of MSCs by encapsulating and transferring many functional factors, including regulatory RNAs, proteins and lipids, however, exosome release is considered a cellular adaptation mechanism and its composition, biogenesis and secretion will depend on microenvironment with which cells interact (Xin et al., 2017a). An example of this cellular adaptation was reported by Harting et al. (2018) in a coculture of MSCs with ischemic tissue extracts, which demonstrated that MSCs can respond to an inflammatory stimulus by producing exosomes with a high anti-inflammatory capacity.

Recent studies show that proteins and regulatory RNAs within MSC-derived exosomes have synergistic effects in crucial processes such as metabolism, neuroinflammation, migration of cellular precursors and processes related to angiogenesis, neurogenesis and synaptogenesis, all activated after injuries (Nakano et al., 2016; Börger et al., 2017; Collino et al., 2017). In a study conducted by Li et al. (2017) in a TBI model, it was reported that dental pulp MSC-derived exosomes alter M1 microglia polarization and promote the transition to M2 phenotype. The M1/M2 transition inhibits the proinflammatory activity of M1 and increases M2 production of anti-inflammatory factors, which decreases neuroinflammation and promotes the functional recovery of rodents; however, the mechanisms that mediates these events remains unknown (Xin et al., 2013a; Doeppner et al., 2015; Li et al., 2017). Nakano et al. (2016) showed that neurological alterations caused by streptozotocin are restored by administration of MSC-derived exosomes, nevertheless, it was reported that there was no generation of new neurons, instead, these vesicles restore and protect the function of remaining neurons by increasing neuritic density and inhibiting oxidative stress damage, mainly lipid peroxidation of neuronal membranes.

In the last years, different studies demonstrated that MSC-derived exosomes promoted neurogenesis in different mice models of disease (Xin et al., 2013b; Doeppner et al., 2015; Zhang Y. et al., 2017). In these studies, treatment with exosomes increased the number of new-born neurons in neurogenic niches (the subventricular zone (SVZ) and dentate gyrus (DG)). However, the concrete cellular and molecular mechanism of this neurogenic process still unclear.

This demonstrates the multimodal therapeutic capabilities of the MSC-derived exosomes as MSC paracrine activity effectors, although the mechanisms remain unknown.

### MSC-Derived Exosomes miRNAs

As mentioned above, exosomes can transfer different RNAs to adjacent cells. Among RNAs, miRNAs are the most widely studied (Cheng et al., 2018). miRNAs are a class of non-coding RNAs that functionally inhibit their respective messenger RNAs target by binding to the 3′ untranslated regions (3′ UTR) and are implicated in many biological processes such as embryonic development, proliferation, differentiation and apoptosis (Stevanato et al., 2016). It has been described that approximately 60% of genes are more than 1,000 miRNAs targets, and 70% of those miRNAs are expressed in the brain, where they regulate different neural and glial functions (Lei et al., 2015). Also, it was demonstrated that the proportion of miRNA is higher in exosomes than in their parent cells (Zhang et al., 2015). The number and type of miRNA within the exosomes is not a random process, instead, the cells selectively group the miRNAs, however, the process of packing RNAs into exosomes is poorly understood (Stevanato et al., 2016). Nevertheless, there are potential ways of sorting miRNAs into exosomes like the neural sphingomyelinase 2, the miRNA induced silencing complex and the miRNA motif sumoylation pathways, however, the underlying mechanisms remain unclear (Zhang et al., 2015).

Several *in vitro* and *in vivo* studies indicate that MSC exosomes transfer functional miRNAs to neural cells and promote neuritic remodeling and plasticity, as well as inhibit apoptosis, which subsequently promotes functional recovery (Xin et al., 2013b, 2017b; Cheng et al., 2018). Few studies have identified a single exosome cargo component that contributes to observed effects (Börger et al., 2017). For example, Xin et al. (2017b) demonstrated that exosomes enriched with miR-133b promote neurovascular plasticity and also reported that this miRNA increases secondary release of exosomes from astrocytes, which considerably enhances neuritic growth, however, they do not exclude the possibility that other cells are influenced by miR-133b. Baglio et al. (2015) analyzed MSC miRNA profiles of bone marrow and adipose tissue, among these miRNAs, there are some that are involved in MSC biology, such as miR-486 that regulates cellular senescence, or miR-143 with a key role in MSC immune response modulation, additionally, other miRNAs were identified, such as miR-191, miR-222, miR-21 and let-7a related to cell cycle progression, proliferation and angiogenesis modulation (Chen et al., 2010; Clark et al., 2014; Baglio et al., 2015).

On the other hand, it has been reported that exosomes also contain miR-98, miR-155 and miR-125a which have antiapoptotic activity (Ma et al., 2016; Cheng et al., 2018). Cheng et al. (2018), showed that in chronic inflammation and apoptotic conditions, miR-21 levels decrease considerably, however, MSCs in this condition secrete exosomes with high levels of miR21, which reduce apoptosis of cells that are in an environment of chronic inflammation. Furthermore, they demonstrated that miR-21 can bind to messenger RNA 3′ UTR of PTEN, main inhibitor of the PI3K/Akt survival pathway in apoptosis mediated by p53 and phosphatidylinositol. Therefore miR-21 possibly promotes cell survival by inhibiting PTEN during apoptosis, triggering the activation of Akt and Bcl-2 and the decrease of Bad, Bax and caspase-3, eventually inhibiting apoptosis.

The miRNA-miR-17-92 cluster, formed by miR-17, miR-18a, miR-19a, miR-19b, miR-20a and miR-92a, has shown to be implicated in neuritic remodeling and neurogenesis as established by Xin et al. (2017a). This cluster, like miR21, targets PTEN, allowing the activation of Akt and mTOR, which phosphorylate GSK-3β, inhibiting its function. GSK-3β inactivation has been reported to stimulate axonal growth and central nervous system recovery (Eldar-Finkelman and Martinez, 2011; Xin et al., 2017a). Moreover, it has been described that MSC exosomes assist in neural differentiation by miR-124 delivering to neural precursor cells (NPCs). This miRNA suppresses Sox9 expression, implied in NPC multipotent capacity and maintenance, hence the effect of miR-124 on Sox9 promotes NPC differentiation (Lee et al., 2014; Yang J. et al., 2017).

Understanding miRNA-regulated molecular mechanisms and their impact on the brain can likely be translated into therapies with positive clinical impact for AD and other neurodegenerative disorders in the future.

### MSC-Derived Exosomes Proteins

Similar to miRNAs, proteins from exosomal cargo are important effectors of these vesicles. Currently, more than 900 proteins have been identified within MSC-derived exosomes (Kalra et al., 2012; Keerthikumar et al., 2016). Exosomal proteins can act as signaling molecules, receptors, cell adhesion molecules among other functions. For example, the expression of proteins such as nestin, neuro-D, growth-associated protein 43, synaptophysins, VEGF, FGF promote events such as neural development, synaptogenesis and angiogenesis (Chopp and Li, 2002). Katsuda et al. (2013) indicated that MSC exosomes from adipose tissue contain neprilysin, an enzyme capable of degrading Aβ, and in co-culture with cells designed for Aβ exacerbated production, these exosomes significantly reduced levels of Aβ1–40 and 1–42.

In different neurodegenerative disease models, it has been reported that MSCs interact with NPC in neurogenic niches of SVZ of lateral ventricles and the hippocampus DG through exosomes (Lee et al., 2013; Zhang and Chopp, 2015; Xin et al., 2017b; Yang Y. et al., 2017). However, the mechanisms by which exosomes interact with NPC and modify their behavior to promote neurogenesis, among other neuroplastic events, have not yet been determined. Nevertheless, some authors have associated some components with the activation (see Table 1), of the chemokine ligand (motif cc) 2 (CCL2), that functions as a neuronal activity modulator. MSCs release CCL2 to stimulate proliferation, migration and differentiation of NPC to neural and glial cells (Liu et al., 2007; Lee et al., 2013).

**Table 1:** Polypeptides identified in exosomes derivate from Mesenchymal Stem Cells (MSC).

Protein name	Gene	UniProtKB^a^ Acc. No.	MW^b^ (kDa)	pI^C^
1. C-C motif chemokine 2 (CCL2)	*CCL2*	P13500	11.02	9.40
2. NAD-dependent protein deacetylase sirtuin-1	*SIRT1*	Q96EB6	81.68	4.55
3. Protein Wnt-3a	*WNT3A*	P56704	39.36	8.52
4. Pentraxin-related protein PTX3	*PTX3*	P26022	41.97	4.94
5. Thrombospondin-1	*THBS1*	P07996	129.38	4.71
6. Growth/differentiation factor 15	*GDF15*	Q99988	34.14	9.79
7. Cell division control protein 42	*CDC42*	P60953	21.25	6.16
8. Dihydropyrimidinase-related protein 2	*DPYSL2*	Q17555	62.29	5.95
9. Prosaposin	*PSAP*	P07602	58.11	5.06
10. Brain-derived neurotrophic factor	*BDNF*	P23560	27.81	9.01
11. Nerve growth factor	*NGF*	P01138	26.95	9.94
12. Fibroblast growth factor 2	*FGF2*	P09038	30.77	11.18
13. Stromal cell-derived factor 1	*CXCL12*	P48061	10.66	9.92
14. Ephrin A-2	*EFNA2*	O43921	23.87	6.99
15. Vascular endothelial growth factor	*VEGFA*	P15692	27.04	9.21
16. Microtubule-associated protein tau	*MAPT*	P10636	78.92	6.25
17. Beta-secretase 1	*BACE1*	P56817	55.76	5.31
18. Amyloid-beta A4 protein	*APP*	P05067	86.94	4.73
19. Prion protein	*PRNP*	P04156	27.66	9.13
20. CD81	*CD81*	P60033	25.80	5.09
21. Tetraspanin-6	*TSPAN6*	O43657	27.56	8.44
22. CD9	*CD9*	P21926	25.41	6.80
23. Neutral sphingomyelinase 2	*SMPD3*	Q9NY59	71.03	5.52

*^a^UniProtKB Acc. Numb., UniProt Knowledgebase Accession Number. ^b^MW, Molecular weight. ^C^pI, Isoelectric point*.

Another identified component is Sirtuin1 (SIRT1), which regulates transcription factors and cofactor deacetylation involved in angiogenesis, inflammation, response to oxidative stress and in neural development, associated with NPC proliferation and differentiation (Hu et al., 2014). SIRT1 forms a complex with Hairy/enhancer of Split 1 (Hes1), a transcriptional repressor of Mash1, responsible for the activation of neuronal specific transcription program. Under oxidizing conditions, this SIRT1/Hes1 complex deacetylates Mash1 promoter and recruits other co-repressors such as TLE1, which block neuronal differentiation, whereas under reducing conditions the SIRT1/Hes1 complex is not formed, therefore Hes1 recruits transcriptional activators such as the CREB binding protein to the Mash1 promoter, resulting in a neural destiny of NPC (Libert et al., 2008).

McBride et al. (2017) found that MSC-derived exosomes transport Wnt3a proteins associated with the outer face of the exosomal membrane. This allows the activation of the Wnt/β catenin signaling pathway, the main canonical signaling process that regulates adult neurogenesis (Yin et al., 2007). It has been reported that this signaling increases in the hippocampus DG after the administration of MSC in TBI models and improves cognitive deficits, as a result of potentiation of neurogenesis. It has been described that this signaling increases in hippocampus DG after MSC administration in TBI models and improves cognitive deficits, associated with the potentiation of neurogenesis (Zhao et al., 2016a; McBride et al., 2017). Wnt3a and its active form β-catenin expression promote NPC expansion and differentiation into synaptically active neurons, whereas the absence of Wnt3a inhibits the differentiation of NPC to neurons (Yin et al., 2007).

Rodriguez-Grande et al. (2015) studied the effect of Pentraxin 3 (PTX3) protein on neurogenesis using a stroke model and reported that PTX3 is a key regulator of angiogenesis and neurogenesis, however, the molecular mechanisms involved have not been described yet. PTX3 is a protein with direct involvement in neuroinflammation in acute phases (Ummenthum et al., 2016). The inhibition of PTX3 reduces the number of capillaries in reperfusion areas after ischemia as well as the formation of new neurons (Rodriguez-Grande et al., 2015).

In the exosomal cargo, ephrins, are a pivotal regulator of the developmental process of axon guidance, cell migration, synapse formation and vascular formation but it is unknown the role they play in the adult organism (Wilkinson, 2001), to this account Holmberg et al. (2005) studied the role of A-class ephrins in the neural stem cell niche, and reported that ephrin-A2 (EFNA2) negatively regulates neural progenitor proliferation. Lack of expression EFNA2 and its receptor Eph7A result in active and ongoing neurogenesis, suggesting that neural cell replacement therapies may be achieved by modification of ephrin signaling pathways.

Dihydropyrimidinase-like 2 (DPYSL2) best known as collapsing response mediator protein 2 also is found in the exosomal cargo. DPYSL2 is a member of a family named for their roles in axonal growth cone collapse. Its main function is stabilizing microtubules, promoting neuritic outgrowth and modulating signaling processes (Pham et al., 2016). In the process of NPC senescence, the expression of DPYSL2 decreases with the age, consistent with the involvement in the neurodegeneration processes (Wang et al., 2016).

Prosaposin (PSAP) is another protein found in exosomes (Li et al., 2010). PSAP is suggested to be an essential neurotrophic factor since its secretion stimulates proliferation and maturation of immature neurons in the hippocampus DG, as well as provides protection against apoptosis. It was reported that deficiency of PSAP precedes massive neuronal loss in neurotoxic environments (Morishita et al., 2014; Nabeka et al., 2017).

It has been recently demonstrated that THBS1 is present in the secretome of MSC and exosomes (Maumus et al., 2017). Blake et al. (2008) show that thrombospondin-1 (THBS1) is a physiological ligand for ApoER2 like Reelin. This study demonstrated that the first alternative physiological ligand for ApoER2 and VLDLR is capable of inducing Dab1 phosphorylation, but no other key events of the Reelin signaling pathway. Blake et al. (2008) also showed that THBS1 increases the length of neuronal precursor chains and stabilizes the structure of established chains along the rostral migratory stream. These functions of THBS1 in neuronal migration could help replace neural cells in injured zones and ameliorate neurological deficits through the administration of exosomes.

**Figure 1:**
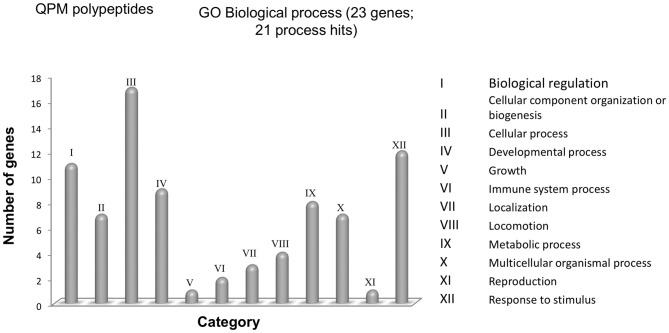
Functional classification with PANTHER of the polypeptides found in exosomes. The UniProtKB IDs of proteins were submitted to the PANTHER database for their classification in Gene Ontology (GO) according to Biological. *X*-axis, categories of proteins. *Y*-axis, number of genes contained in each category.

An analysis of a bioinformatic database was performed in order to identify and classify exosomal cargo of MSC according to their biologic function and their interaction in the secretome. The 23 proteins described in Table 1 were classified by Protein Analysis Through Evolutionary Relationships (PANTHER) system (Mi et al., 2017) and were grouped according to their involvement in the different cellular biological processes. In this first approach, we found 12 different biological processes (Figure 1; a protein can participate in more than one cellular process). From these 12 biological processes, four main groups are mentioned as; (a) cellular processes with 17 members; (b) response to a stimulus with 12 members; (c) biological regulation with 11 members; and (d) development processes with nine members.

In the cellular processes group, there are 13 proteins involved in cellular communication and four proteins with a role in the movement of cellular components. The main proteins implicated in cellular communication are members of CXC chemokine family such as CCL2 (UniProt code P13500) and CXCL2 (UniProt code P19875; The UniProt Consortium, 2018). A recent work in murine models of neurodegeneration has associated these two proteins in cellular migration processes and enhanced proliferation and differentiation of neural precursors (Hong et al., 2015; Wang F. et al., 2017). In addition, another member of this family, CXCR4 expressed by neurons (UniProt code P61073) has been linked to inflammatory processes by activating microglia expressing CCR2 (UniProt code P41597; Liu C. et al., 2014). One study showed that knockout of CCR2 in an AD transgenic mouse model decreases microglia activation and increases Aβ accumulation (Kiyota et al., 2013). This demonstrates the role of microglia in Aβ clearance and how its deficiency could speed up AD progression.

The second most important biological process was response to stimuli, mainly the regulation of protein phosphorylation, where the neurotrophic factors VEGF (UniProt code P15692), NGF (UniProt code P01138) and BDNF (UniProt code P23560) that modulate cell death cascades, increase production of proteins responsible for proliferation and maintenance of neurons. These factors also have roles in the outgrowth of dendrites and stabilizing synapses between neurons. In recent years, these neurotrophins have been considered as key regulators of adult neurogenesis and the changes in expression have been related to occurrence and development of cognitive impairments, even though the molecular mechanism is not completely elucidated (Ke and Zhang, 2013; Budni et al., 2015; Vilar and Mira, 2016). However, more data and support are needed to elucidate the mechanisms of neurotrophin imbalance and dysregulation in AD as well as possible therapeutic applications.

On the other hand, the main molecular functions identified for these molecules are related to catalytic activity, signal transduction and protein binding. In these cases, protein binding activity is the most representative molecular function for 12 proteins implied. In this group neurotrophins can also be found, due to their activity, which is mediated mainly by receptor phosphorylation which subsequently promotes the expression of proteins involved in the proliferation of the NPC, maintenance of the cell and ensuring neuronal survival (Bolijn and Lucassen, 2015).

**Figure 2:**
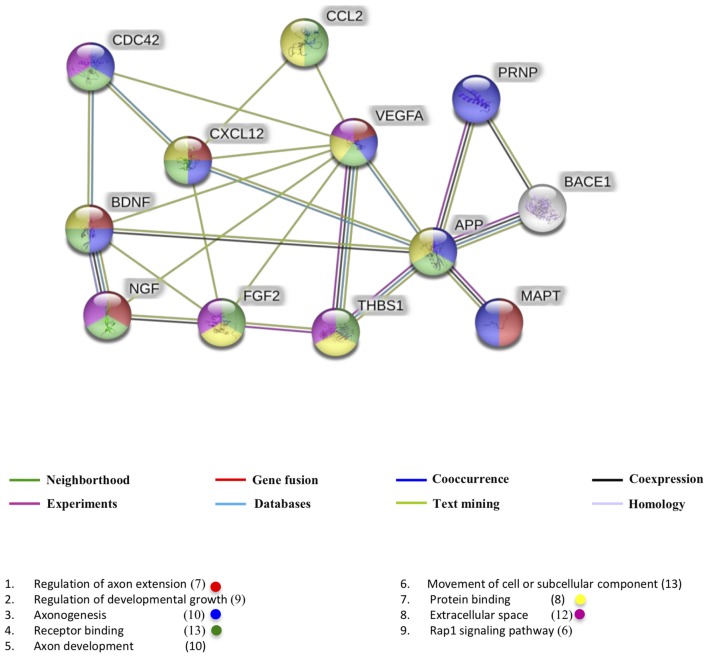
Interactome of polypeptides found in common exosomes related with a beta and tau protein. UniProtKB accession numbers were submitted to the String program to identify the predicted functional network. Lines in color represent different pieces of evidence for each identified interaction: red line, fusion; green line, neighborhood; blue line, cooccurrence; purple line, experimental; yellow line, text mining; light blue line, database; black line, coexpression.

This classification allowed us to generate a network of known and predicted protein-protein interaction using the STRING program (Szklarczyk et al., 2017). The interactome network represented in Figure 2 describes the interactome with a minimum required interaction score of 0.70 (high confidence) and highlights the biological processes in the regulation of axon extension (shown in red) with seven members in it and a false discovery rate (FDR) of 4.78e^−09^.

The second most important process for our analysis is axonogenesis with 10 members and an FDR of 8.91e^−08^, shown in blue. Interesting members related to axonogenesis are tau (MAPT UniProt code P10636) and cell division control protein 42 (CDC42 UniProt code P60953). It is known that tau is accumulated in the growth cone and its presence persists during the axonal elongation, however, understand the role of tau in axonogenesis is complicated because tau exists in different phosphorylation states and these states influence the subsequent localization of tau within neurons without implication of its role in the progression of AD (Zmuda and Rivas, 2000). CDC42 has roles in axon guidance and neurite formation particularly on growth cone through Robo signaling activation and actin filaments regulation (Matsuura et al., 2004). The CXCL12 and the neurotrophins BDNF and NGF are also associated with axonogenesis. Almost all proteins exert their function by acting as ligands (shown in green with an FDR 4.02e^−08^).

The proteins of interactome network are usually found in the extracellular space (shown in pink with an FDR 1.8e^−06^) where they can modulate the processes like the responses to stimuli previously described. The main pathway of this interactome network was the Rap1 signaling pathway (FDR 2.3e^−05^) which has been reported to regulate vesicle secretion, cytoskeletal dynamics, proliferation and cell adhesion, (Shibasaki et al., 2007; van Hooren et al., 2012; Zhang Y.-L. et al., 2017). Possibly this way of signaling supports the delivery of the exosomal cargo.

On the other hand, it is interesting that VEGF participates in all analyzed processes. It has been reported that this neurotrophic factor evokes elements of brain plasticity like neurogenesis and neural progenitor cells migration (Chen et al., 2005). According to the interaction diagram, VEGF has synergistic effects with some neurotrophins and with components that mediate axonal guidance such as CDC42 and THBS1 (UniProt code P07996). This leads us to think that possibly the synergy of the exosomal cargo promotes better therapeutic responses compared to those that a single isolated component could. It would be important to study the effects of the composition of the exosomal charge on the progression of AD in both interactions with Aβ and the tau protein, as well as the effects it could have on neuroplastic events, mainly neurogenesis and synaptogenesis.

## Conclusion and Perspectives

Despite the great advances in AD research, the molecular mechanisms underlying this devastating disease have not been fully unveiled. However, remarkable neuropathological studies have provided the largest contribution to the knowledge of the mechanisms involved in the pathological amyloidogenic processing of Aβ as well as hyperphosphorylated tau aggregation into paired helical filaments. Unfortunately, there remains a need to find an accurate diagnosis, in addition to generating really effective treatments; thus, it is necessary to use novel approaches to understand the molecular and cellular mechanisms of AD in order to identify new therapeutic strategies that allow to delay, reverse or, in the best case, to avoid the normal pathological processing of this disease.

The use of proteomic study technologies plus the advent of induced pluripotent stem cells and three-dimensional culture technologies, has made it possible to generate novel *in vitro* 3D neural cell culture models that replicate AD pathologies, allowing us to explore new perspectives on the origin of the disease and its progression, for example the influence of some proteins in the misfolding of Aβ and the tau protein and its resistance to degradation. These *in vitro* 3D neural cell culture models also could explore the biochemical composition and modulation of exosomes and their role in disease progression. These advances have revolutionized the potential to generate novel platforms that can be used to study the mechanisms of pathology or to develop novel diagnostic and therapeutic tools in a brain-like environment.

Currently, MSC therapy has emerged as a promising strategy for treating different neurodegenerative disorders via tissue repair, however, the risks of tumor formation, cellular rejection and thrombosis in MSCs transplantation remain unresolved. Currently, the cell-free therapy using MSC-derived exosomes might constitute an alternative because of their advantages over MSCs. There are different studies indicating that exosomes act as an important mediator of the information exchange between MSCs and NPC. The exchange of miRNA and proteins between cell to cell through exosomes can reduce the neuroinflammation, promote neurogenesis and angiogenesis rescue learning impairments and improve functional recovery. However, the concrete mechanisms involved in the positive effects induced by MSCs-derived exosomes in AD are still unclear. Given a variety of functions and multiple molecules in exosomal cargo, is necessary that other studies analyze all interaction and understand the relation between the intrinsic potential that is glimpsed in the combination of the use of exosome therapy and the participation of their cargo (miRNA and/or proteins) combined with the proteomic and bioinformatic analysis of those pathways that participate in this therapeutic modulation.

The bioinformatic analysis performed, allowed us to focus on possible candidates with an important role in neurogenesis and neuroplasticity or even identify some potential pathways implicated in AD’s patient’s progress. This would allow us to use exosomes with different therapeutic approaches, for example, the modification of exosomes with some classes of proteins or miRNAs, with effects on tissue repair, maintenance of cellular homeostasis or impairing the disease progression.

## Author Contributions

ER-Z, MH-S, BM, YG-M, AM-A and AC-A: equal contribution for the literature search, writing and correcting of this review article.

## Conflict of Interest Statement

The authors declare that the research was conducted in the absence of any commercial or financial relationships that could be construed as a potential conflict of interest.

## References

[B1] Abels E. R. Breakefield X. O. (2016). Introduction to extracellular vesicles: biogenesis, RNA cargo selection, content, release, and uptake. Cell. Mol. Neurobiol. 36, 301–312. 10.1007/s10571-016-0366-z 27053351 PMC5546313

[B2] Angeloni N. L. McMahon K. M. Swaminathan S. Plebanek M. P. Osman I. Volpert O. V.. (2016). Pathways for modulating exosome lipids identified by high-density lipoprotein-like nanoparticle binding to scavenger receptor type B-1. Sci. Rep. 6:22915. 10.1038/srep22915 26964503 PMC4786789

[B3] Baglio S. R. Rooijers K. Koppers-Lalic D. Verweij F. J. Pérez Lanzón M. Zini N.. (2015). Human bone marrow- and adipose-mesenchymal stem cells secrete exosomes enriched in distinctive miRNA and tRNA species. Stem Cell Res. Ther. 6:127. 10.1186/s13287-015-0116-z 26129847 PMC4529699

[B4] Bang C. Thum T. (2012). Exosomes: new players in cell-cell communication. Int. J. Biochem. Cell Biol. 44, 2060–2064. 10.1016/j.biocel.2012.08.007 22903023

[B5] Baumann K. Mandelkow E.-M. Biernat J. Piwnica-Worms H. Mandelkow E. (1993). Abnormal Alzheimer-like phosphorylation of tau-protein by cyclin-dependent kinases cdk2 and cdk5. FEBS Lett. 336, 417–424. 10.1016/0014-5793(93)80849-p 8282104

[B6] Beer K. B. Wehman A. M. (2017). Mechanisms and functions of extracellular vesicle release *in vivo*—What we can learn from flies and worms. Cell Adh. Migr. 11, 135–150. 10.1080/19336918.2016.1236899 27689411 PMC5351733

[B7] Blake S. M. Strasser V. Andrade N. Duit S. Hofbauer R. Schneider W. J.. (2008). Thrombospondin-1 binds to ApoER2 and VLDL receptor and functions in postnatal neuronal migration. EMBO J. 27, 3069–3080. 10.1038/emboj.2008.223 18946489 PMC2585172

[B8] Bolijn S. Lucassen P. J. (2015). How the body talks to the brain; peripheral mediators of physical activity-induced proliferation in the adult hippocampus. Brain Plasticity 1, 5–27. 10.3233/bpl-150020 29765833 PMC5939189

[B9] Börger V. Bremer M. Ferrer-Tur R. Gockeln L. Stambouli O. Becic A.. (2017). Mesenchymal stem/stromal cell-derived extracellular vesicles and their potential as novel immunomodulatory therapeutic agents. Int. J. Mol. Sci. 18:E1450. 10.3390/ijms18071450 28684664 PMC5535941

[B10] Braak H. Braak E. (1996). Evolution of the neuropathology of Alzheimer’s disease. Acta Neurol. Scand. Suppl. 165, 3–12. 10.1111/j.1600-0404.1996.tb05866.x 8740983

[B11] Budni J. Bellettini-Santos T. Mina F. Garcez M. L. Zugno A. I. (2015). The involvement of BDNF, NGF and GDNF in aging and Alzheimer’s disease. Aging Dis. 6, 331–341. 10.14336/ad.2015.0825 26425388 PMC4567216

[B12] Camacho L. Guerrero P. Marchetti D. (2013). MicroRNA and protein profiling of brain metastasis competent cell-derived exosomes. PLoS One 8:e73790. 10.1371/journal.pone.0073790 24066071 PMC3774795

[B13] Cantinieaux D. Quertainmont R. Blacher S. Rossi L. Wanet T. Noël A.. (2013). Conditioned medium from bone marrow-derived mesenchymal stem cells improves recovery after spinal cord injury in rats: an original strategy to avoid cell transplantation. PLoS One 8:e69515. 10.1371/journal.pone.0069515 24013448 PMC3754952

[B16] Chen T. S. Lai R. C. Lee M. M. Choo A. B. Lee C. N. Lim S. K. (2010). Mesenchymal stem cell secretes microparticles enriched in pre-microRNAs. Nucleic Acids Res. 38, 215–224. 10.1093/nar/gkp857 19850715 PMC2800221

[B14] Chen J. Zhang C. Jiang H. Li Y. Zhang L. Robin A.. (2005). Atorvastatin induction of VEGF and BDNF promotes brain plasticity after stroke in mice. J. Cereb. Blood Flow Metab. 25, 281–290. 10.1038/sj.jcbfm.9600034 15678129 PMC2804085

[B15] Chen J. J. Zhao B. Zhao J. Li S. (2017). Potential roles of exosomal micrornas as diagnostic biomarkers and therapeutic application in Alzheimer’s disease. Neural Plast. 2017:7027380. 10.1155/2017/7027380 28770113 PMC5523215

[B17] Cheng L. Wu S. Zhang K. Qing Y. Xu T. (2017). A comprehensive overview of exosomes in ovarian cancer: emerging biomarkers and therapeutic strategies. J. Ovarian Res. 10:73. 10.1186/s13048-017-0368-6 29100532 PMC5670635

[B18] Cheng X. Zhang G. Zhang L. Hu Y. Zhang K. Sun X.. (2018). Mesenchymal stem cells deliver exogenous miR-21 via exosomes to inhibit nucleus pulposus cell apoptosis and reduce intervertebral disc degeneration. J. Cell. Mol. Med. 22, 261–276. 10.1111/jcmm.13316 28805297 PMC5742691

[B19] Chiarini A. Armato U. Gardenal E. Gui L. Dal Prà I. (2017). Amyloid β-exposed human astrocytes overproduce phospho-tau and overrelease it within exosomes, effects suppressed by calcilytic NPS 2143-further implications for Alzheimer’s therapy. Front. Neurosci. 11:217. 10.3389/fnins.2017.00217 28473749 PMC5397492

[B20] Cho H. Choi J. Y. Hwang M. S. Kim Y. J. Lee H. M. Lee H. S.. (2016). *In vivo* cortical spreading pattern of tau and amyloid in the Alzheimer disease spectrum. Ann. Neurol. 80, 247–258. 10.1002/ana.24711 27323247

[B21] Chopp M. Li Y. (2002). Treatment of neural injury with marrow stromal cells. Lancet Neurol. 1, 92–100. 10.1016/s1474-4422(02)00040-6 12849513

[B22] Clark E. A. Kalomoiris S. Nolta J. A. Fierro F. A. (2014). Concise review: MicroRNA function in multipotent mesenchymal stromal cells. Stem Cells 32, 1074–1082. 10.1002/stem.1623 24860868 PMC10668871

[B23] Collino F. Pomatto M. Bruno S. Lindoso R. S. Tapparo M. Sicheng W.. (2017). Exosome and microvesicle-enriched fractions isolated from mesenchymal stem cells by gradient separation showed different molecular signatures and functions on renal tubular epithelial cells. Stem Cell Rev. 13, 226–243. 10.1007/s12015-016-9713-1 28070858 PMC5380712

[B24] Colombo M. Raposo G. Théry C. (2014). Biogenesis, secretion, and intercellular interactions of exosomes and other extracellular vesicles. Annu. Rev. Cell Dev. Biol. 30, 255–289. 10.1146/annurev-cellbio-101512-122326 25288114

[B25] Correas I. Díaz-Nido J. Avila J. (1992). Microtubule-associated protein tau is phosphorylated by protein kinase C on its tubulin binding domain. J. Biol. Chem. 267, 15721–15728. 1639808

[B26] de Jong O. G. Verhaar M. C. Chen Y. Vader P. Gremmels H. Posthuma G.. (2012). Cellular stress conditions are reflected in the protein and RNA content of endothelial cell-derived exosomes. J. Extracell. Vesicles 1:18396. 10.3402/jev.v1i0.18396 24009886 PMC3760650

[B27] Dinkins M. B. Enasko J. Hernandez C. Wang G. Kong J. Helwa I.. (2016). Neutral sphingomyelinase-2 deficiency ameliorates Alzheimer’s disease pathology and improves cognition in the 5XFAD mouse. J. Neurosci. 36, 8653–8667. 10.1523/JNEUROSCI.1429-16.2016 27535912 PMC4987436

[B28] Doeppner T. R. Herz J. Görgens A. Schlechter J. Ludwig A. K. Radtke S.. (2015). Extracellular vesicles improve post-stroke neuroregeneration and prevent postischemic immunosuppression. Stem Cells Transl. Med. 4, 1131–1143. 10.5966/sctm.2015-0078 26339036 PMC4572905

[B29] Donders R. Bogie J. F. J. Ravanidis S. Gervois P. Vanheusden M. Marée R.. (2018). Human wharton’s jelly-derived stem cells display a distinct immunomodulatory and proregenerative transcriptional signature compared to bone marrow-derived stem cells. Stem Cells Dev. 27, 65–84. 10.1089/scd.2017.0029 29267140

[B30] Drommelschmidt K. Serdar M. Bendix I. Herz J. Bertling F. Prager S.. (2017). Mesenchymal stem cell-derived extracellular vesicles ameliorate inflammation-induced preterm brain injury. Brain Behav. Immun. 60, 220–232. 10.1016/j.bbi.2016.11.011 27847282

[B31] Eitan E. Suire C. Zhang S. Mattson M. P. (2016). Impact of lysosome status on extracellular vesicle content and release. Ageing Res. Rev. 32, 65–74. 10.1016/j.arr.2016.05.001 27238186 PMC5154730

[B32] Eldar-Finkelman H. Martinez A. (2011). GSK-3 inhibitors: preclinical and clinical focus on CNS. Front. Mol. Neurosci. 4:32. 10.3389/fnmol.2011.00032 22065134 PMC3204427

[B33] Emmanouilidou E. Melachroinou K. Roumeliotis T. Garbis S. D. Ntzouni M. Margaritis L. H.. (2010). Cell-produced α-synuclein is secreted in a calcium-dependent manner by exosomes and impacts neuronal survival. J. Neurosci. 30, 6838–6851. 10.1523/JNEUROSCI.5699-09.2010 20484626 PMC3842464

[B34] Escola J. M. Kleijmeer M. J. Stoorvogel W. Griffith J. M. Yoshie O. Geuze H. J. (1998). Selective enrichment of tetraspan proteins on the internal vesicles of multivesicular endosomes and on exosomes secreted by human B-lymphocytes. J. Biol. Chem. 273, 20121–20127. 10.1074/jbc.273.32.20121 9685355

[B35] Falker C. Hartmann A. Guett I. Dohler F. Altmeppen H. Betzel C.. (2016). Exosomal cellular prion protein drives fibrillization of amyloid β and counteracts amyloid β-mediated neurotoxicity. J. Neurochem. 137, 88–100. 10.1111/jnc.13514 26710111

[B36] Gauthier S. A. Pérez-González R. Sharma A. Huang F. K. Alldred M. J. Pawlik M.. (2017). Enhanced exosome secretion in Down syndrome brain—a protective mechanism to alleviate neuronal endosomal abnormalities. Acta Neuropathol. Commun. 5:65. 10.1186/s40478-017-0466-0 28851452 PMC5576289

[B37] Ghosh A. Giese K. P. (2015). Calcium/calmodulin-dependent kinase II and Alzheimer’s disease. Mol. Brain 8:78. 10.1186/s13041-015-0166-2 26603284 PMC4657223

[B38] Goetzl E. J. Mustapic M. Kapogiannis D. Eitan E. Lobach I. V. Goetzl L.. (2016). Cargo proteins of plasma astrocyte-derived exosomes in Alzheimer’s disease. FASEB J. 30, 3853–3859. 10.1096/fj.201600756r 27511944 PMC5067254

[B39] Gomzikova M. O. Rizvanov A. A. (2017). Current trends in regenerative medicine: from cell to cell-free therapy. BioNanoScience 7, 240–245. 10.1007/s12668-016-0348-0

[B40] Greenberg S. M. Koo E. H. Selkoe D. J. Qiu W. Q. Kosik K. S. (1994). Secreted β-amyloid precursor protein stimulates mitogen-activated protein kinase and enhances tau phosphorylation. Proc. Natl. Acad. Sci. U S A 91, 7104–7108. 10.1073/pnas.91.15.7104 8041753 PMC44347

[B41] Grundke-Iqbal I. Iqbal K. Tung Y. C. Quinlan M. Wisniewski H. M. Binder L. I. (1986). Abnormal phosphorylation of the microtubule-associated protein tau (tau) in Alzheimer cytoskeletal pathology. Proc. Natl. Acad. Sci. U S A 83, 4913–4917. 10.1073/pnas.83.13.4913 3088567 PMC323854

[B42] Guduric-Fuchs J. O’Connor A. Camp B. O’Neill C. L. Medina R. J. Simpson D. A. (2012). Selective extracellular vesicle-mediated export of an overlapping set of microRNAs from multiple cell types. BMC Genomics 13:357. 10.1186/1471-2164-13-357 22849433 PMC3532190

[B43] Guix F. X. Sannerud R. Berditchevski F. Arranz A. M. Horré K. Snellinx A.. (2017). Tetraspanin 6: a pivotal protein of the multiple vesicular body determining exosome release and lysosomal degradation of amyloid precursor protein fragments. Mol. Neurodegener. 12:25. 10.1186/s13024-017-0165-0 28279219 PMC5345265

[B44] Hall J. Prabhakar S. Balaj L. Lai C. P. Cerione R. A. Breakefield X. O. (2016). Delivery of therapeutic proteins via extracellular vesicles: review and potential treatments for Parkinson’s disease, glioma, and schwannoma. Cell. Mol. Neurobiol. 36, 417–427. 10.1007/s10571-015-0309-0 27017608 PMC4860146

[B45] Harris V. K. Stark J. Vyshkina T. Blackshear L. Joo G. Stefanova V.. (2018). Phase I trial of intrathecal mesenchymal stem cell-derived neural progenitors in progressive multiple sclerosis. EBioMedicine 29, 23–30. 10.1016/j.ebiom.2018.02.002 29449193 PMC5925446

[B46] Harting M. T. Srivastava A. K. Zhaorigetu S. Bair H. Prabhakara K. S. Toledano Furman N. E.. (2018). Inflammation-stimulated mesenchymal stromal cell-derived extracellular vesicles attenuate inflammation. Stem Cells 36, 79–90. 10.1002/stem.2730 29076623

[B47] Henne W. M. Stenmark H. Emr S. D. (2013). Molecular mechanisms of the membrane sculpting ESCRT pathway. Cold Spring Harb. Perspect. Biol. 5:a016766. 10.1101/cshperspect.a016766 24003212 PMC3753708

[B48] Holmberg J. Armulik A. Senti K.-A. Edoff K. Spalding K. Momma S.. (2005). Ephrin-A2 reverse signaling negatively regulates neural progenitor proliferation and neurogenesis. Genes Dev. 19, 462–471. 10.1101/gad.326905 15713841 PMC548947

[B49] Hong Y. R. Lee H. Park M. H. Lee J. K. Lee J. Y. Suh H. D.. (2015). CCL2 induces neural stem cell proliferation and neuronal differentiation in Niemann-Pick type C mice. J. Vet. Med. Sci. 77, 693–699. 10.1292/jvms.14-0352 25715651 PMC4488406

[B50] Hu B. Guo Y. Chen C. Li Q. Niu X. Guo S.. (2014). Repression of SIRT1 promotes the differentiation of mouse induced pluripotent stem cells into neural stem cells. Cell. Mol. Neurobiol. 34, 905–912. 10.1007/s10571-014-0071-8 24832395 PMC11488914

[B51] Hurley J. H. Hanson P. I. (2010). Membrane budding and scission by the ESCRT machinery: it’s all in the neck. Nat. Rev. Mol. Cell Biol. 11, 556–566. 10.1038/nrm2937 20588296 PMC2922035

[B52] Ikezu T. Ikezu S. Varnum M. Wolozin B. Butovsky O. Kügler S. (2016). Microglial exosomes propagate tau protein from the entorhinal cortex to the hippocampus: an early pathophysiology of Alzheimer’s disease. Alzheimers Dement. 12, P339–P340. 10.1016/j.jalz.2016.06.624

[B53] Jaber N. Mohd-Naim N. Wang Z. DeLeon J. L. Kim S. Zhong H.. (2016). Vps34 regulates Rab7 and late endocytic trafficking through recruitment of the GTPase-activating protein Armus. J. Cell Sci. 129, 4424–4435. 10.1242/jcs.192260 27793976 PMC5201010

[B54] Jaber V. Zhao Y. Lukiw W. J. (2017). Alterations in micro RNA-messenger RNA (miRNA-mRNA) coupled signaling networks in sporadic Alzheimer’s disease (AD) hippocampal CA1. J. Alzheimers Dis. Parkinsonism 7:312. 10.4172/2161-0460.1000312 29051843 PMC5645033

[B55] Joshi P. Benussi L. Furlan R. Ghidoni R. Verderio C. (2015). Extracellular vesicles in Alzheimer’s disease: friends or foes? Focus on Aβ-vesicle interaction. Int. J. Mol. Sci. 16, 4800–4813. 10.3390/ijms16034800 25741766 PMC4394450

[B56] Kalra H. Simpson R. J. Ji H. Aikawa E. Altevogt P. Askenase P.. (2012). Vesiclepedia: a compendium for extracellular vesicles with continuous community annotation. PLoS Biol. 10:e1001450. 10.1371/journal.pbio.1001450 23271954 PMC3525526

[B57] Katsuda T. Tsuchiya R. Kosaka N. Yoshioka Y. Takagaki K. Oki K.. (2013). Human adipose tissue-derived mesenchymal stem cells secrete functional neprilysin-bound exosomes. Sci. Rep. 3:1197. 10.1038/srep01197 23378928 PMC3561625

[B58] Ke X.-J. Zhang J.-J. (2013). Changes in HIF-1α, VEGF, NGF and BDNF levels in cerebrospinal fluid and their relationship with cognitive impairment in patients with cerebral infarction. J. Huazhong Univ. Sci. Technolog. Med. Sci. 33, 433–437. 10.1007/s11596-013-1137-4 23771673

[B59] Keerthikumar S. Chisanga D. Ariyaratne D. Al Saffar H. Anand S. Zhao K.. (2016). ExoCarta: a web-based compendium of exosomal cargo. J. Mol. Biol. 428, 688–692. 10.1016/j.jmb.2015.09.019 26434508 PMC4783248

[B60] Kessels H. W. Nguyen L. N. Nabavi S. Malinow R. (2010). The prion protein as a receptor for amyloid-β. Nature 466, E3–E4; discussion E4–E5. 10.1038/nature09217 20703260 PMC3057871

[B61] Kim H. J. Lee J. H. Kim S. H. (2010). Therapeutic effects of human mesenchymal stem cells on traumatic brain injury in rats: secretion of neurotrophic factors and inhibition of apoptosis. J. Neurotrauma 27, 131–138. 10.1089/neu.2008.0818 19508155

[B62] Kiyota T. Gendelman H. E. Weir R. A. Higgins E. E. Zhang G. Jain M. (2013). CCL2 affects β-amyloidosis and progressive neurocognitive dysfunction in a mouse model of Alzheimer’s disease. Neurobiol. Aging 34, 1060–1068. 10.1016/j.neurobiolaging.2012.08.009 23040664 PMC4011558

[B63] Kizuka Y. Kitazume S. Fujinawa R. Saito T. Iwata N. Saido T. C.. (2015). An aberrant sugar modification of BACE1 blocks its lysosomal targeting in Alzheimer’s disease. EMBO Mol. Med. 7, 175–189. 10.15252/emmm.201404438 25592972 PMC4328647

[B64] Koelsch G. (2017). BACE1 function and inhibition: implications of intervention in the amyloid pathway of Alzheimer’s disease pathology. Molecules 22:E1723. 10.3390/molecules22101723 29027981 PMC6151801

[B65] Kosaka N. Iguchi H. Yoshioka Y. Takeshita F. Matsuki Y. Ochiya T. (2010). Secretory mechanisms and intercellular transfer of microRNAs in living cells. J. Biol. Chem. 285, 17442–17452. 10.1074/jbc.M110.107821 20353945 PMC2878508

[B66] Kosik K. S. Joachim C. L. Selkoe D. J. (1986). Microtubule-associated protein tau (tau) is a major antigenic component of paired helical filaments in Alzheimer disease. Proc. Natl. Acad. Sci. U S A 83, 4044–4048. 10.1073/pnas.83.11.4044 2424016 PMC323662

[B67] Kurozumi K. Nakamura K. Tamiya T. Kawano Y. Kobune M. Hirai S.. (2004). BDNF gene-modified mesenchymal stem cells promote functional recovery and reduce infarct size in the rat middle cerebral artery occlusion model. Mol. Ther. 9, 189–197. 10.1016/j.ymthe.2003.10.012 14759803

[B68] Lai R. C. Arslan F. Lee M. M. Sze N. S. Choo A. Chen T. S.. (2010). Exosome secreted by MSC reduces myocardial ischemia/reperfusion injury. Stem Cell Res. 4, 214–222. 10.1016/j.scr.2009.12.003 20138817

[B69] Laulagnier K. Javalet C. Hemming F. J. Chivet M. Lachenal G. Blot B.. (2018). Amyloid precursor protein products concentrate in a subset of exosomes specifically endocytosed by neurons. Cell. Mol. Life Sci. 75, 757–773. 10.1007/s00018-017-2664-0 28956068 PMC11105273

[B70] Lauren J. Gimbel D. A. Nygaard H. B. Gilbert J. W. Strittmatter S. M. (2009). Cellular prion protein mediates impairment of synaptic plasticity by amyloid-β oligomers. Nature 457, 1128–1132. 10.1038/nature07761 19242475 PMC2748841

[B72] Lee H. K. Finniss S. Cazacu S. Xiang C. Brodie C. (2014). Mesenchymal stem cells deliver exogenous miRNAs to neural cells and induce their differentiation and glutamate transporter expression. Stem Cells Dev. 23, 2851–2861. 10.1089/scd.2014.0146 25036385

[B71] Lee H. Kang J. E. Lee J. K. Bae J. S. Jin H. K. (2013). Bone-marrow-derived mesenchymal stem cells promote proliferation and neuronal differentiation of Niemann-Pick type C mouse neural stem cells by upregulation and secretion of CCL2. Hum. Gene Ther. 24, 655–669. 10.1089/hum.2013.001 23659480 PMC3719464

[B73] Lei X. Lei L. Zhang Z. Zhang Z. Cheng Y. (2015). Downregulated miR-29c correlates with increased BACE1 expression in sporadic Alzheimer’s disease. Int. J. Clin. Exp. Pathol. 8, 1565–1574. 25973041 PMC4396232

[B74] Levy E. (2017). Exosomes in the diseased brain: first insights from *in vivo* studies. Front. Neurosci. 11:142. 10.3389/fnins.2017.00142 28386213 PMC5362612

[B77] Li Y. Chen J. Chen X. G. Wang L. Gautam S. C. Xu Y. X.. (2002). Human marrow stromal cell therapy for stroke in rat: neurotrophins and functional recovery. Neurology 59, 514–523. 10.1212/wnl.59.4.514 12196642

[B76] Li Y. Kim J. (2016). CB2 cannabinoid receptor knockout in mice impairs contextual long-term memory and enhances spatial working memory. Neural Plast. 2016:9817089. 10.1155/2016/9817089 26819779 PMC4706977

[B75] Li N. Sarojini H. An J. Wang E. (2010). Prosaposin in the secretome of marrow stroma-derived neural progenitor cells protects neural cells from apoptotic death. J. Neurochem. 112, 1527–1538. 10.1111/j.1471-4159.2009.06565.x 20050969

[B78] Li Y. Yang Y. Y. Ren J. L. Xu F. Chen F. M. Li A. (2017). Exosomes secreted by stem cells from human exfoliated deciduous teeth contribute to functional recovery after traumatic brain injury by shifting microglia M1/M2 polarization in rats. Stem Cell Res. Ther. 8:198. 10.1186/s13287-017-0648-5 28962585 PMC5622448

[B79] Libert S. Cohen D. Guarente L. (2008). Neurogenesis directed by Sirt1. Nat. Cell Biol. 10, 373–374. 10.1038/ncb0408-373 18379594 PMC2703710

[B80] Liu C. Cui G. Zhu M. Kang X. Guo H. (2014). Neuroinflammation in Alzheimer’s disease: chemokines produced by astrocytes and chemokine receptors. Int. J. Clin. Exp. Pathol. 7, 8342–8355. 25674199 PMC4314046

[B81] Liu C. G. Song J. Zhang Y. Q. Wang P. C. (2014). MicroRNA-193b is a regulator of amyloid precursor protein in the blood and cerebrospinal fluid derived exosomal microRNA-193b is a biomarker of Alzheimer’s disease. Mol. Med. Rep. 10, 2395–2400. 10.3892/mmr.2014.2484 25119742

[B82] Liu X. S. Zhang Z. G. Zhang R. L. Gregg S. R. Wang L. Yier T.. (2007). Chemokine ligand 2 (CCL2) induces migration and differentiation of subventricular zone cells after stroke. J. Neurosci. Res. 85, 2120–2125. 10.1002/jnr.21359 17510981

[B83] Lugli G. Cohen A. M. Bennett D. A. Shah R. C. Fields C. J. Hernandez A. G.. (2015). Plasma exosomal miRNAs in persons with and without alzheimer disease: altered expression and prospects for biomarkers. PLoS One 10:e0139233. 10.1371/journal.pone.0139233 26426747 PMC4591334

[B84] Luna-Muñoz J. Chávez-Macías L. García-Sierra F. Mena R. (2007). Earliest stages of tau conformational changes are related to the appearance of a sequence of specific phospho-dependent tau epitopes in Alzheimer’s disease. J. Alzheimers Dis. 12, 365–375. 10.3233/jad-2007-12410 18198423

[B85] Ma J. F. Zang L. N. Xi Y. M. Yang W. J. Zou D. (2016). MiR-125a Rs12976445 polymorphism is associated with the apoptosis status of nucleus pulposus cells and the risk of intervertebral disc degeneration. Cell. Physiol. Biochem. 38, 295–305. 10.1159/000438630 26800505

[B86] Malm T. Loppi S. Kanninen K. M. (2016). Exosomes in Alzheimer’s disease. Neurochem. Int. 97, 193–199. 10.1016/j.neuint.2016.04.011 27131734

[B87] Mandelkow E. M. Drewes G. Biernat J. Gustke N. Van Lint J. Vandenheede J. R.. (1992). Glycogen synthase kinase-3 and the Alzheimer-like state of microtubule-associated protein tau. FEBS Lett. 314, 315–321. 10.1016/0014-5793(92)81496-9 1334849

[B88] Matsuura R. Tanaka H. Go M. J. (2004). Distinct functions of Rac1 and Cdc42 during axon guidance and growth cone morphogenesis in *Drosophila*. Eur. J. Neurosci. 19, 21–31. 10.1046/j.1460-9568.2003.03084.x 14750960

[B89] Matthay M. A. Pati S. Lee J. W. (2017). Concise review: mesenchymal stem (Stromal) cells: biology and preclinical evidence for therapeutic potential for organ dysfunction following trauma or sepsis. Stem Cells 35, 316–324. 10.1002/stem.2551 27888550

[B200] Maumus M. Manferdini C. Toupet K. Chuchana P. Casteilla L. Gachet M.. (2017). Thrombospondin-1 partly mediates the cartilage protective effect of adipose-derived mesenchymal stem cells in osteoarthritis. Front Immunol. 8:1638. 10.3389/fimmu.2017.01638 29238343 PMC5712679

[B90] McBride J. D. Rodriguez-Menocal L. Guzman W. Candanedo A. Garcia-Contreras M. Badiavas E. V. (2017). Bone marrow mesenchymal stem cell-derived CD63^+^ exosomes transport Wnt3a exteriorly and enhance dermal fibroblast proliferation, migration and angiogenesis *in vitro*. Stem Cells Dev. 26, 1384–1398. 10.1089/scd.2017.0087 28679315

[B91] Mi H. Huang X. Muruganujan A. Tang H. Mills C. Kang D.. (2017). PANTHER version 11: expanded annotation data from Gene Ontology and Reactome pathways and data analysis tool enhancements. Nucleic Acids Res. 45, D183–D189. 10.1093/nar/gkw1138 27899595 PMC5210595

[B92] Miranda A. M. Lasiecka Z. M. Xu Y. Neufeld J. Shahriar S. Simoes S.. (2018). Neuronal lysosomal dysfunction releases exosomes harboring APP C-terminal fragments and unique lipid signatures. Nat. Commun. 9:291. 10.1038/s41467-017-02533-w 29348617 PMC5773483

[B93] Mitsialis S. A. Kourembanas S. (2016). Stem cell-based therapies for the newborn lung and brain: possibilities and challenges. Semin. Perinatol. 40, 138–151. 10.1053/j.semperi.2015.12.002 26778234 PMC4808378

[B94] Mizushima N. Komatsu M. (2011). Autophagy: renovation of cells and tissues. Cell 147, 728–741. 10.1016/j.cell.2011.10.026 22078875

[B95] Morishita M. Nabeka H. Shimokawa T. Miyawaki K. Doihara T. Saito S.. (2014). Temporal changes in prosaposin expression in the rat dentate gyrus after birth. PLoS One 9:e95883. 10.1371/journal.pone.0095883 24871372 PMC4037173

[B96] Mroczko B. Groblewska M. Litman-Zawadzka A. Kornhuber J. Lewczuk P. (2018). Amyloid β oligomers (AβOs) in Alzheimer’s disease. J. Neural Transm. 125, 177–191. 10.1007/s00702-017-1820-x 29196815

[B97] Mulcahy L. A. Pink R. C. Carter D. R. (2014). Routes and mechanisms of extracellular vesicle uptake. J. Extracell. Vesicles 3:24641. 10.3402/jev.v3.24641 25143819 PMC4122821

[B98] Munro K. M. Nash A. Pigoni M. Lichtenthaler S. F. Gunnersen J. M. (2016). Functions of the Alzheimer’s disease protease BACE1 at the synapse in the central nervous system. J. Mol. Neurosci. 60, 305–315. 10.1007/s12031-016-0800-1 27456313 PMC5059407

[B99] Nabeka H. Saito S. Li X. Shimokawa T. Khan M. S. I. Yamamiya K.. (2017). Interneurons secrete prosaposin, a neurotrophic factor, to attenuate kainic acid-induced neurotoxicity. IBRO Rep. 3, 17–32. 10.1016/j.ibror.2017.07.001 30135939 PMC6084830

[B100] Nakano M. Nagaishi K. Konari N. Saito Y. Chikenji T. Mizue Y.. (2016). Bone marrow-derived mesenchymal stem cells improve diabetes-induced cognitive impairment by exosome transfer into damaged neurons and astrocytes. Sci. Rep. 6:24805. 10.1038/srep24805 27102354 PMC4840335

[B101] Nath S. Agholme L. Kurudenkandy F. R. Granseth B. Marcusson J. Hallbeck M. (2012). Spreading of neurodegenerative pathology via neuron-to-neuron transmission of β-amyloid. J. Neurosci. 32, 8767–8777. 10.1523/JNEUROSCI.0615-12.2012 22745479 PMC6622335

[B102] Nguyen T. M. Arthur A. Hayball J. D. Gronthos S. (2013). EphB and Ephrin-B interactions mediate human mesenchymal stem cell suppression of activated T-cells. Stem Cells Dev. 22, 2751–2764. 10.1089/scd.2012.0676 23711177 PMC3787464

[B103] Nikitidou E. Khoonsari P. E. Shevchenko G. Ingelsson M. Kultima K. Erlandsson A. (2017). Increased release of apolipoprotein E in extracellular vesicles following amyloid-β protofibril exposure of neuroglial co-cultures. J. Alzheimers Dis. 60, 305–321. 10.3233/jad-170278 28826183 PMC5676865

[B104] Ophelders D. R. Wolfs T. G. Jellema R. K. Zwanenburg A. Andriessen P. Delhaas T.. (2016). Mesenchymal stromal cell-derived extracellular vesicles protect the fetal brain after hypoxia-ischemia. Stem Cells Transl. Med. 5, 754–763. 10.5966/sctm.2015-0197 27160705 PMC4878333

[B105] Ostrowski M. Carmo N. B. Krumeich S. Fanget I. Raposo G. Savina A.. (2009). Rab27a and Rab27b control different steps of the exosome secretion pathway. Nat. Cell Biol. 12, 19–30. 10.1038/ncb2000 19966785

[B106] Pham X. Song G. Lao S. Goff L. Zhu H. Valle D.. (2016). The DPYSL2 gene connects mTOR and schizophrenia. Transl. Psychiatry 6:e933. 10.1038/tp.2016.204 27801893 PMC5314117

[B107] Phinney D. G. Pittenger M. F. (2017). Concise review: MSC-derived exosomes for cell-free therapy. Stem Cells 35, 851–858. 10.1002/stem.2575 28294454

[B108] Piper R. C. Katzmann D. J. (2007). Biogenesis and function of multivesicular bodies. Annu. Rev. Cell Dev. Biol. 23, 519–547. 10.1146/annurev.cellbio.23.090506.123319 17506697 PMC2911632

[B109] Qu J. Zhang H. (2017). Roles of mesenchymal stem cells in spinal cord injury. Stem Cells Int. 2017:5251313. 10.1155/2017/5251313 28630630 PMC5467343

[B110] Rajendran L. Honsho M. Zahn T. R. Keller P. Geiger K. D. Verkade P.. (2006). Alzheimer’s disease β-amyloid peptides are released in association with exosomes. Proc. Natl. Acad. Sci. U S A 103, 11172–11177. 10.1073/pnas.0603838103 16837572 PMC1544060

[B111] Reiman E. M. Quiroz Y. T. Fleisher A. S. Chen K. Velez-Pardo C. Jimenez-Del-Rio M.. (2012). Brain imaging and fluid biomarker analysis in young adults at genetic risk for autosomal dominant Alzheimer’s disease in the presenilin 1 E280A kindred: a case-control study. Lancet Neurol. 11, 1048–1056. 10.1016/S1474-4422(12)70228-4 23137948 PMC4181671

[B112] Reiss A. B. Arain H. A. Stecker M. M. Siegart N. M. Kasselman L. J. (2018). Amyloid toxicity in Alzheimer’s disease. Rev. Neurosci. 29, 613–627. 10.1515/revneuro-2017-0063 29447116

[B113] Rodriguez-Grande B. Varghese L. Molina-Holgado F. Rajkovic O. Garlanda C. Denes A.. (2015). Pentraxin 3 mediates neurogenesis and angiogenesis after cerebral ischaemia. J. Neuroinflammation 12:15. 10.1186/s12974-014-0227-y 25616391 PMC4308938

[B114] Saman S. Kim W. Raya M. Visnick Y. Miro S. Saman S.. (2012). Exosome-associated tau is secreted in tauopathy models and is selectively phosphorylated in cerebrospinal fluid in early Alzheimer disease. J. Biol. Chem. 287, 3842–3849. 10.1074/jbc.M111.277061 22057275 PMC3281682

[B115] Saman S. Lee N. C. Inoyo I. Jin J. Li Z. Doyle T.. (2014). Proteins recruited to exosomes by tau overexpression implicate novel cellular mechanisms linking tau secretion with Alzheimer’s disease. J. Alzheimers Dis. 40, S47–S70. 10.3233/jad-132135 24718102 PMC5977388

[B116] Scott C. W. Spreen R. C. Herman J. L. Chow F. P. Davison M. D. Young J.. (1993). Phosphorylation of recombinant tau by cAMP-dependent protein kinase. Identification of phosphorylation sites and effect on microtubule assembly. J. Biol. Chem. 268, 1166–1173. 8419321

[B117] Sharples R. A. Vella L. J. Nisbet R. M. Naylor R. Perez K. Barnham K. J.. (2008). Inhibition of γ-secretase causes increased secretion of amyloid precursor protein C-terminal fragments in association with exosomes. FASEB J. 22, 1469–1478. 10.1096/fj.07-9357com 18171695

[B118] Shi M. Kovac A. Korff A. Cook T. J. Ginghina C. Bullock K. M.. (2016). CNS tau efflux via exosomes is likely increased in Parkinson’s disease but not in Alzheimer’s disease. Alzheimers Dement. 12, 1125–1131. 10.1016/j.jalz.2016.04.003 27234211 PMC5107127

[B119] Shibasaki T. Takahashi H. Miki T. Sunaga Y. Matsumura K. Yamanaka M.. (2007). Essential role of Epac2/Rap1 signaling in regulation of insulin granule dynamics by cAMP. Proc. Natl. Acad. Sci. U S A 104, 19333–19338. 10.1073/pnas.0707054104 18040047 PMC2148290

[B120] Smith P. Y. Hernandez-Rapp J. Jolivette F. Lecours C. Bisht K. Goupil C.. (2015). miR-132/212 deficiency impairs tau metabolism and promotes pathological aggregation *in vivo*. Hum. Mol. Genet. 24, 6721–6735. 10.1093/hmg/ddv377 26362250 PMC4634376

[B121] Spencer B. Kim C. Gonzalez T. Bisquertt A. Patrick C. Rockenstein E.. (2016). α-Synuclein interferes with the ESCRT-III complex contributing to the pathogenesis of Lewy body disease. Hum. Mol. Genet. 25, 1100–1115. 10.1093/hmg/ddv633 26740557 PMC4764192

[B122] Stevanato L. Thanabalasundaram L. Vysokov N. Sinden J. D. (2016). Investigation of content, stoichiometry and transfer of miRNA from human neural stem cell line derived exosomes. PLoS One 11:e0146353. 10.1371/journal.pone.0146353 26752061 PMC4713432

[B123] Stoothoff W. H. Johnson G. V. (2005). Tau phosphorylation: physiological and pathological consequences. Biochim. Biophys. Acta 1739, 280–297. 10.1016/j.bbadis.2004.06.017 15615646

[B124] Stuffers S. Sem Wegner C. Stenmark H. Brech A. (2009). Multivesicular endosome biogenesis in the absence of ESCRTs. Traffic 10, 925–937. 10.1111/j.1600-0854.2009.00920.x 19490536

[B125] Svensson K. J. Christianson H. C. Wittrup A. Bourseau-Guilmain E. Lindqvist E. Svensson L. M.. (2013). Exosome uptake depends on ERK1/2-heat shock protein 27 signaling and lipid Raft-mediated endocytosis negatively regulated by caveolin-1. J. Biol. Chem. 288, 17713–17724. 10.1074/jbc.M112.445403 23653359 PMC3682571

[B126] Szklarczyk D. Morris J. H. Cook H. Kuhn M. Wyder S. Simonovic M.. (2017). The STRING database in 2017: quality-controlled protein-protein association networks, made broadly accessible. Nucleic Acids Res. 45, D362–D368. 10.1093/nar/gkw937 27924014 PMC5210637

[B127] Takahashi R. H. Milner T. A. Li F. Nam E. E. Edgar M. A. Yamaguchi H.. (2002). Intraneuronal Alzheimer Aβ42 accumulates in multivesicular bodies and is associated with synaptic pathology. Am. J. Pathol. 161, 1869–1879. 10.1016/s0002-9440(10)64463-x 12414533 PMC1850783

[B128] Tan J. Evin G. (2012). β-site APP-cleaving enzyme 1 trafficking and Alzheimer’s disease pathogenesis. J. Neurochem. 120, 869–880. 10.1111/j.1471-4159.2011.07623.x 22171895

[B129] Tang Y. Bao J. S. Su J. H. Huang W. (2017). MicroRNA-139 modulates Alzheimer’s-associated pathogenesis in SAMP8 mice by targeting cannabinoid receptor type 2. Genet. Mol. Res. 16, 10–4238. 10.4238/gmr16019166 28218780

[B134] The UniProt Consortium. (2018). UniProt: the universal protein knowledgebase. Nucleic Acids Res. 46, 2699–2699. 10.1093/nar/gky092 29425356 PMC5861450

[B130] Timmers L. Lim S. K. Arslan F. Armstrong J. S. Hoefer I. E. Doevendans P. A.. (2007). Reduction of myocardial infarct size by human mesenchymal stem cell conditioned medium. Stem Cell Res. 1, 129–137. 10.1016/j.scr.2008.02.002 19383393

[B131] Trajkovic K. Hsu C. Chiantia S. Rajendran L. Wenzel D. Wieland F.. (2008). Ceramide triggers budding of exosome vesicles into multivesicular endosomes. Science 319, 1244–1247. 10.1126/science.1153124 18309083

[B132] Trotta T. Panaro M. A. Cianciulli A. Mori G. Di Benedetto A. Porro C. (2018). Microglia-derived extracellular vesicles in Alzheimer’s disease: a double-edged sword. Biochem. Pharmacol. 148, 184–192. 10.1016/j.bcp.2017.12.020 29305855

[B133] Ummenthum K. Peferoen L. A. Finardi A. Baker D. Pryce G. Mantovani A.. (2016). Pentraxin-3 is upregulated in the central nervous system during MS and EAE, but does not modulate experimental neurological disease. Eur. J. Immunol. 46, 701–711. 10.1002/eji.201545950 26576501

[B135] van Hooren K. W. van Agtmaal E. L. Fernandez-Borja M. van Mourik J. A. Voorberg J. Bierings R. (2012). The Epac-Rap1 signaling pathway controls cAMP-mediated exocytosis of Weibel-Palade bodies in endothelial cells. J. Biol. Chem. 287, 24713–24720. 10.1074/jbc.M111.321976 22511766 PMC3397898

[B136] Vella L. J. Sharples R. A. Nisbet R. M. Cappai R. Hill A. F. (2008). The role of exosomes in the processing of proteins associated with neurodegenerative diseases. Eur. Biophys. J. 37, 323–332. 10.1007/s00249-007-0246-z 18064447

[B137] Vilar M. Mira H. (2016). Regulation of neurogenesis by neurotrophins during adulthood: expected and unexpected roles. Front. Neurosci. 10:26. 10.3389/fnins.2016.00026 26903794 PMC4746328

[B138] Villarroya-Beltri C. Gutiérrez-Vázquez C. Sánchez-Cabo F. Pérez-Hernández D. Vázquez J. Martin-Cofreces N.. (2013). Sumoylated hnRNPA2B1 controls the sorting of miRNAs into exosomes through binding to specific motifs. Nat. Commun. 4:2980. 10.1038/ncomms3980 24356509 PMC3905700

[B139] Vingtdeux V. Sergeant N. Buee L. (2012). Potential contribution of exosomes to the prion-like propagation of lesions in Alzheimer’s disease. Front. Physiol. 3:229. 10.3389/fphys.2012.00229 22783199 PMC3389776

[B140] Wakabayashi T. Craessaerts K. Bammens L. Bentahir M. Borgions F. Herdewijn P.. (2009). Analysis of the γ-secretase interactome and validation of its association with tetraspanin-enriched microdomains. Nat. Cell Biol. 11, 1340–1346. 10.1038/ncb1978 19838174

[B141] Wang F. Baba N. Shen Y. Yamashita T. Tsuru E. Tsuda M.. (2017). CCL11 promotes migration and proliferation of mouse neural progenitor cells. Stem Cell Res. Ther. 8:26. 10.1186/s13287-017-0474-9 28173860 PMC5297016

[B144] Wang Y. Balaji V. Kaniyappan S. Krüger L. Irsen S. Tepper K.. (2017). The release and trans-synaptic transmission of Tau via exosomes. Mol. Neurodegener. 12:5. 10.1186/s13024-016-0143-y 28086931 PMC5237256

[B142] Wang G. Dinkins M. He Q. Zhu G. Poirier C. Campbell A.. (2012). Astrocytes secrete exosomes enriched with proapoptotic ceramide and prostate apoptosis response 4 (PAR-4): potential mechanism of apoptosis induction in Alzheimer disease (AD). J. Biol. Chem. 287, 21384–21395. 10.1074/jbc.M112.340513 22532571 PMC3375560

[B143] Wang X. Dong C. Sun L. Zhu L. Sun C. Ma R.. (2016). Quantitative proteomic analysis of age-related subventricular zone proteins associated with neurodegenerative disease. Sci. Rep. 6:37443. 10.1038/srep37443 27857231 PMC5114652

[B145] Wei X. Yang X. Han Z. P. Qu F. F. Shao L. Shi Y. F. (2013). Mesenchymal stem cells: a new trend for cell therapy. Acta Pharmacol. Sin. 34, 747–754. 10.1038/aps.2013.50 23736003 PMC4002895

[B146] Wilkinson D. G. (2001). Multiple roles of EPH receptors and ephrins in neural development. Nat. Rev. Neurosci. 2, 155–164. 10.1038/35058515 11256076

[B147] Wischik C. M. Crowther R. A. Stewart M. Roth M. (1985). Subunit structure of paired helical filaments in Alzheimer’s disease. J. Cell Biol. 100, 1905–1912. 10.1083/jcb.100.6.1905 2581978 PMC2113596

[B148] Wischik C. M. Novak M. Edwards P. C. Klug A. Tichelaar W. Crowther R. A. (1988). Structural characterization of the core of the paired helical filament of Alzheimer disease. Proc. Natl. Acad. Sci. U S A 85, 4884–4888. 10.1073/pnas.85.13.4884 2455299 PMC280541

[B149] Wu Y. Deng W. Klinke D. J. II. (2015). Exosomes: improved methods to characterize their morphology, RNA content, and surface protein biomarkers. Analyst 140, 6631–6642. 10.1039/c5an00688k 26332016 PMC4986832

[B150] Xiao T. Zhang W. Jiao B. Pan C. Z. Liu X. Shen L. (2017). The role of exosomes in the pathogenesis of Alzheimer’ disease. Transl. Neurodegener. 6:3. 10.1186/s40035-017-0072-x 28184302 PMC5289036

[B151] Xin H. Katakowski M. Wang F. Qian J. Y. Liu X. S. Ali M. M.. (2017a). MicroRNA cluster miR-17–92 cluster in exosomes enhance neuroplasticity and functional recovery after stroke in rats. Stroke 48, 747–753. 10.1161/STROKEAHA.116.015204 28232590 PMC5330787

[B154] Xin H. Wang F. Li Y. Lu Q. E. Cheung W. L. Zhang Y.. (2017b). Secondary release of exosomes from astrocytes contributes to the increase in neural plasticity and improvement of functional recovery after stroke in rats treated with exosomes harvested from MicroRNA 133b-overexpressing multipotent mesenchymal stromal cells. Cell Transplant. 26, 243–257. 10.3727/096368916x693031 27677799 PMC5303172

[B152] Xin H. Li Y. Cui Y. Yang J. J. Zhang Z. G. Chopp M. (2013a). Systemic administration of exosomes released from mesenchymal stromal cells promote functional recovery and neurovascular plasticity after stroke in rats. J. Cereb. Blood Flow Metab. 33, 1711–1715. 10.1038/jcbfm.2013.152 23963371 PMC3824189

[B153] Xin H. Li Y. Liu Z. Wang X. Shang X. Cui Y.. (2013b). MiR-133b promotes neural plasticity and functional recovery after treatment of stroke with multipotent mesenchymal stromal cells in rats via transfer of exosome-enriched extracellular particles. Stem Cells 31, 2737–2746. 10.1002/stem.1409 23630198 PMC3788061

[B155] Xiong Y. Mahmood A. Chopp M. (2017). Emerging potential of exosomes for treatment of traumatic brain injury. Neural Regen. Res. 12, 19–22. 10.4103/1673-5374.198966 28250732 PMC5319225

[B156] Yan R. Fan Q. Zhou J. Vassar R. (2016). Inhibiting BACE1 to reverse synaptic dysfunctions in Alzheimer’s disease. Neurosci. Biobehav. Rev. 65, 326–340. 10.1016/j.neubiorev.2016.03.025 27044452 PMC4856578

[B158] Yang Y. Ye Y. Su X. He J. Bai W. He X. (2017). MSCs-derived exosomes and neuroinflammation, neurogenesis and therapy of traumatic brain injury. Front. Cell. Neurosci. 11:55. 10.3389/fncel.2017.00055 28293177 PMC5329010

[B157] Yang J. Zhang X. Chen X. Wang L. Yang G. (2017). Exosome mediated delivery of miR-124 promotes neurogenesis after ischemia. Mol. Ther. Nucleic Acids 7, 278–287. 10.1016/j.omtn.2017.04.010 28624203 PMC5415550

[B159] Yin Z. S. Zhang H. Wang W. Hua X. Y. Hu Y. Zhang S. Q.. (2007). Wnt-3a protein promote neuronal differentiation of neural stem cells derived from adult mouse spinal cord. Neurol. Res. 29, 847–854. 10.1179/016164107x223539 17609021

[B160] Yuyama K. Igarashi Y. (2017). Exosomes as carriers of Alzheimer’s amyloid-ß. Front. Neurosci. 11:229. 10.3389/fnins.2017.00229 28487629 PMC5403946

[B161] Zappulli V. Friis K. P. Fitzpatrick Z. Maguire C. A. Breakefield X. O. (2016). Extracellular vesicles and intercellular communication within the nervous system. J. Clin. Invest. 126, 1198–1207. 10.1172/jci81134 27035811 PMC4811121

[B166] Zhang Z. G. Chopp M. (2015). Promoting brain remodeling to aid in stroke recovery. Trends Mol. Med. 21, 543–548. 10.1016/j.molmed.2015.07.005 26278490 PMC4567429

[B164] Zhang Y. Chopp M. Zhang Z. G. Katakowski M. Xin H. Qu C.. (2017). Systemic administration of cell-free exosomes generated by human bone marrow derived mesenchymal stem cells cultured under 2D and 3D conditions improves functional recovery in rats after traumatic brain injury. Neurochem. Int. 111, 69–81. 10.1016/j.neuint.2016.08.003 27539657 PMC5311054

[B163] Zhang J. Li S. Li L. Li M. Guo C. Yao J.. (2015). Exosome and exosomal microRNA: trafficking, sorting, and function. Genomics Proteomics Bioinformatics 13, 17–24. 10.1016/j.gpb.2015.02.001 25724326 PMC4411500

[B165] Zhang Y.-L. Wang R.-C. Cheng K. Ring B. Z. Su L. (2017). Roles of Rap1 signaling in tumor cell migration and invasion. Cancer Biol. Med. 14, 90–99. 10.20892/j.issn.2095-3941.2016.0086 28443208 PMC5365179

[B162] Zhang G. Yang P. (2018). A novel cell-cell communication mechanism in the nervous system: exosomes. J. Neurosci. Res. 96, 45–52. 10.1002/jnr.24113 28718905

[B167] Zhao Y. Gibb S. L. Zhao J. Moore A. N. Hylin M. J. Menge T.. (2016a). Wnt3a, a protein secreted by mesenchymal stem cells is neuroprotective and promotes neurocognitive recovery following traumatic brain injury. Stem Cells 34, 1263–1272. 10.1002/stem.2310 26840479

[B168] Zhao Y. Tan W. Sheng W. Li X. (2016b). Identification of biomarkers associated With Alzheimer’s disease by bioinformatics analysis. Am. J. Alzheimers Dis. Other Demen. 31, 163–168. 10.1177/1533317515588181 26082458 PMC10852637

[B169] Zheng T. Pu J. Chen Y. Mao Y. Guo Z. Pan H.. (2017). Plasma exosomes spread and cluster around β-amyloid plaques in an animal model of Alzheimer’s disease. Front. Aging. Neurosci. 9:12. 10.3389/fnagi.2017.00012 28203202 PMC5285341

[B170] Zmuda J. F. Rivas R. J. (2000). Actin disruption alters the localization of tau in the growth cones of cerebellar granule neurons. J. Cell Sci. 113, 2797–2809. 10.1002/1097-4695(20000615)43:4<313::aid-neu1>3.0.co;2-2 10893194

